# A coupled mathematical model between bone remodeling and tumors: a study of different scenarios using Komarova’s model

**DOI:** 10.1007/s10237-023-01689-3

**Published:** 2023-03-15

**Authors:** Salah Ramtani, Juan Felipe Sánchez, Abdelkader Boucetta, Reuben Kraft, Juan Jairo Vaca-González, Diego A. Garzón-Alvarado

**Affiliations:** 1 Laboratoire CSPBAT, equipe LBPS, CNRS (UMR 7244), Universit e Sorbonne Paris Nord, Paris, France; 2 Biotechnology Institute, Universidad Nacional de Colombia, Bogotá, Colombia; 3 Department of Mechanical Engineering, Penn State University, University Park, USA; 4 Escuela de Pregrado - Direccion Académica, Universidad Nacional de Colombia, Sede de La Paz, Cesar, Colombia

**Keywords:** Bone remodeling, Tumor, Coupling tumor–bone, Komarova’s model, Osteoclasts, Osteoblasts

## Abstract

This paper aims to construct a general framework of coupling tumor–bone remodeling processes in order to produce plausible outcomes of the effects of tumors on the number of osteoclasts, osteoblasts, and the frequency of the bone turnover cycle. In this document, Komarova’s model has been extended to include the effect of tumors on the bone remodeling processes. Thus, we explored three alternatives for coupling tumor presence into Komarova’s model: first, using a “damage” parameter that depends on the tumor cell concentration. A second model follows the original structure of Komarova, including the tumor presence in those equations powered up to a new parameter, called the paracrine effect of the tumor on osteoclasts and osteoblasts; the last model is replicated from Ayati and collaborators in which the impact of the tumor is included into the paracrine parameters. Through the models, we studied their stability and considered some examples that can reproduce the tumor effects seen in clinic and experimentally. Therefore, this paper has three parts: the exposition of the three models, the results and discussion (where we explore some aspects and examples of the solution of the models), and the conclusion.

## Introduction

Understanding bone physiology is crucial in grasping the correct operation of the whole metabolic process in humans and, in general, all species. Bone metabolism is essential in the regulation of minerals, proteins, and hormones. Remarkably, all the processes related to bone turnover due to bone metabolism are produced by a very well-regulated cycle called bone remodeling. This cycle is carried out in a temporal period when a basic multicellular unit (BMU) can make the continuous replacement of bone matrix regulated by two critical cells: osteoblasts and osteoclasts. Bone remodeling occurs permanently in discrete and random locations in the skeleton, both in space and in time, starting with the resorption of the bone matrix by osteoclasts and continuing with the formation of a new structure throughout the osteoblast’s performance (Bonfoh et al. 2008; Martin and Ng 1994; Günther and Schinke 2000; Parfitt 1994).This entire has been studied in the recent period, and it has been shown that the complexity of the process is the result of several key factors that take place in the process itself. This includes continuous operations that provide a survival advantage as the result of coordination between metabolic and mechanical regulators, which affect the BMU activity (Bolamperti et al. 2022). Recent studies have also shown that expansion of osteoprogenitor cell is an integrated process with the resorption, and this expansion is initiated by osteoclasts (Delaisse et al. 2020). This dynamic process behind bone remodeling is modified by mechanical loads, hormones, and pathological processes such as bone cancer and tumors (Burr 2002; Parfitt 2002).

Pathological processes can produce imbalances in the bone remodeling cycle in terms of period, number of cells present in the BMU, bone matrix and quality (Barkaoui et al. 2017). Thus, crucial diseases can produce an imbalance in the bone remodeling cycle: osteoporosis, osteomalacia, osteomyelitis, Paget, bone tumors, and multiple myeloma, among others (Barkaoui et al. 2017). Hence, a better comprehension of the bone remodeling process and the coupling with those diseases allows understanding new perspectives and possibilities for dealing with tumor development treatments and maintaining bone quality.

Certain bone diseases can reduce mass bone, such as multiple myeloma, which is considered as a type of cancer developed in white cells, also known as plasma cells. These cells help the immune system fight against infections by producing antibodies that attack the suspicious elements in the blood system. In this specific disease, cancer cells are accumulated in the bone marrow, reducing the number of healthy blood cells and producing abnormal proteins that cause unbalanced metabolism (Brigle and Rogers 2017). Although the origin of the myeloma has not been completely understood, it is hypothesized that blood cells are formed abnormally in bone marrow, leading to fatigue, fractures, and a malfunctioning of the immune system and bone remodeling cycle (Joshua et al. 2019). This results in antibodies being accumulated in the body, causing kidney damage and reducing bone density (Brigle and Rogers 2017; Joshua et al. 2019). In addition, metastasis commonly presents in the bone due to primary tumors produced in the prostate, colorectal, thyroid, breast, and lung, among others (Fornetti et al. 2018). If primary tumor cells migrate to the bone for making metastasis, the probability of curing the condition is dramatically reduced, and associated morbidities become relevant, i.e., pain, fracture risk and hypercalcemia. In 1889, Stephen Paget hypothesized how tumor cells favor certain organs to boost their spreading, especially the bone (Fornetti et al. 2018). These findings and the close relationship between bone remodeling and bones reveal the importance of understanding the coupling process between tumors and bone turnover. Indeed, the understanding of how tumors and bone are impacted each other remains unclear (Fornetti et al. 2018).

Diseases such as osteosarcoma can produce new abnormal bone mass, corresponding to bone cells that grow excessively and take advantage of bone remodeling process for its expansion. In fact, bone cells have a modification in their DNA, changing the information about how, when, and where these cells should produce new bone, and as a result, those modified cells can invade and destroy healthy tissues (Corre et al. 2020). In addition, primary tumors grow until they produce bone metastasis which will lead to insufficient bone apposition, which in turn is translated into osteolysis with the loss of mechanical properties (Garbayo et al. 2004).

From a computational point of view, Komarova et al. (2003) have established a dynamical system, representing the coupling between the cellular and molecular process from the bone remodeling perspective. Regarding the cellular function, Komarova et al. have proposed the interaction of osteoblasts and osteoclasts (Komarova et al. 2003). The paracrine and autocrine effects on cellular behavior were considered from a molecular perspective. Thereafter, Komarova’s laboratory presented a new mathematical model that is centered on the Parathyroid Hormone, which can allow the regulation of the bone remodeling cycle (Komarova 2006). These previously mentioned works have been explored extensively in bone remodeling field and they have analyzed in order to test multiple scenarios and hypotheses vis-á-vis bone turnover. For instance, Zumsande et al. studied the dynamical system developed by Komorova and its stability (Zumsande et al. 2011); and Ryser et al. extended the Komarova’s model to include a complete list of molecular mechanisms like osteoprotegerin (OPG) and nuclear factor ligand (RANKL) represented in five nonlinear partial differential equations (Ryser et al. 2010). Additionally, Komarova’s model was broadened to include the effect of multiple myeloma disease as presented in (Burr 2002; Ayati et al. 2010). In those papers, the authors found how tumors increased the number of osteoclasts in the bone remodeling process, concluding that the matrix decreased its density.

This paper aims to construct a general framework of coupling tumor–bone remodeling processes in order to plausible results of the effects of tumors on the number of osteoclasts, osteoblasts, and the frequency of the bone turnover cycle. Here, Komarova’s model has been extended to include the effect of tumors on the bone remodeling processes. Thus, we explored three alternatives for coupling tumor presence into Komarova’s model: first, using a “damage” parameter that depends on the tumor cell concentration. A second model follows the original structure of Komarova, including the tumor presence in those equations powered up to a new parameter, called the paracrine effect of the tumor on osteoclasts and osteoblasts; the last model is replicated from (Ayati et al. 2010) in which the impact of the tumor is included into the paracrine parameters. Through the models, we studied their stability and considered some examples that can reproduce the tumor’s clinical and experimental effects observed. Therefore, this paper has three parts: the exposition of the three models, the results and discussion (where we explore some aspects and examples of the solution of the models), and the conclusion. The authors hope this paper will serve as a cornerstone that can then be used as a research framework for those who want to run mathematical models of a coupling tumor–bone remodeling process as a silicon laboratory of possible effects.

## Materials and methods

We used Komarova’s model, which consists of two nonlinear differential equations. These equations are regulated by biochemical factors called paracrine and autocrine (Eq. (1) (Komarova et al. 2003). In addition, one equation for formation and resorption in the system of equations allows quantifying the bone mass in each time step.

1a $$ \frac{\mathrm{d}x_1}{\mathrm{d}t}={\alpha }_1 x^{g_{11}}_1x^{g_{21}}_2-\beta _1x_1 $$

1b $$\begin{aligned} & \frac{\mathrm{d}x_2}{\mathrm{d}t}=\alpha _2 x^{g_{12}}_1x^{g_{22}}_2-\beta _2 x_2\end{aligned}$$

1c $$\begin{aligned} & \frac{\mathrm{d}z}{\mathrm{d}t}={-k}_1y_1+k_2y_2 \end{aligned}$$

where $x_1$ and $x_2$ are the number of osteoclasts and osteoblasts, respectively, $g_{ij}$ is the paracrine factor on the cell *j* due to the presence of *i*-cell, and *z* is the bone mass percentage, that has taken into account the difference between the number of osteoclasts/osteoblasts and its steady state (named $\overline{x_i}$), i.e., ${2y}_i=\left( x_i-\overline{x_i}\right) + \mid \left( x_i-\overline{x_i}\right) \mid$. Three extended models of Komarova’s one are presented here, coupling the bone remodeling model with another equation that considers the tumor growth. The coupling terms used in Komarova’s equation have different structures for each framework and can include more parameters, as shown in the following sections.

**Fig. 1:**
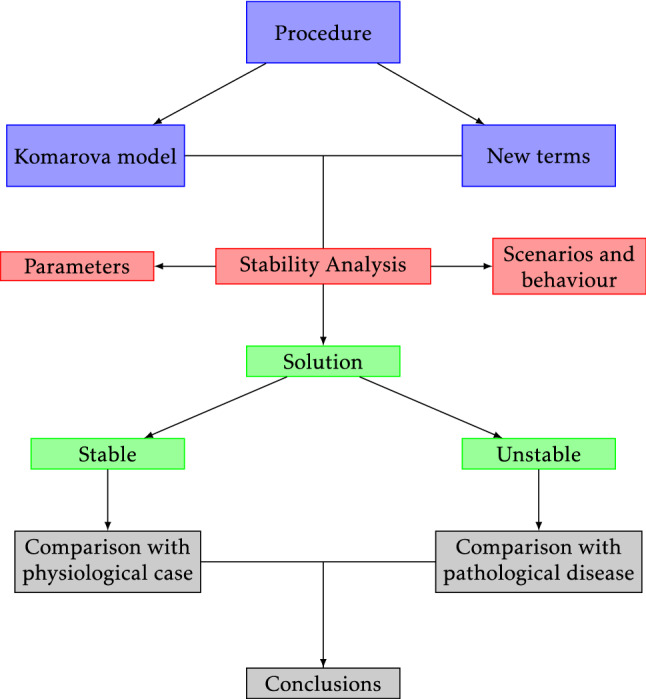
General procedure

The entire process is depicted in diagram in Fig. 1; each color indicates a different stage of the process and how the article is structured. This diagram presents the main steps followed for the modeling process. The procedure consists in using the Komarova’s base model and adding or modifying with new terms the model, to generate a new one. Then, a stability analysis is done. From this analysis, different scenarios and behaviors are obtained for the variation of the different parameters of each model. Having this, solutions are mathematically computed and classified according to their temporal behavior. These behaviors can be compared to the physiological or pathological scenarios in real cases. Finally, some conclusions are shown for the models and their limitations. The entire process is depicted in diagram in Fig. 1, each color indicates a different stage of the process and how the article is structured. This diagram presents the main steps followed for the modeling process. The procedure consists in using the Komarova’s base model and adding or modifying with new terms the model, to generate a new one. Then, a stability analysis is done. From this analysis different scenarios and behaviors are obtained for the variation of the different parameters of each model. Having this, solutions are mathematically computed and classified according to their temporal behavior. These behaviors can be compared to the physiological or pathological scenarios in real cases. Finally, some conclusions are shown for the models and their limitations. The entire process is depicted in diagram in Fig. 1, each color indicates a different stage of the process and how the article is structured. This diagram presents the main steps followed for the modeling process. The procedure consists in using the Komarova’s base model and adding or modifying with new terms the model, to generate a new one. Then a stability analysis is done. From this analysis different scenarios and behaviors are obtained for the variation of the different parameters of each model. Having this, solutions are mathematically computed and classified according to their temporal behavior. These behaviors can be compared to the physiological or pathological scenarios in real cases. Finally, some conclusions are shown for the models and their limitations.

### Tumor affecting the activity of cell production or removal

Komarova’s model is revisited to include a new term or element: the tumor effect on the osteoclasts and osteoblasts rate. In addition, the tumor equation is similar to that given by Ayati et al. (2010). Accordingly, the original differential equations are modified, and it is included the tumor rate as follows:

2a $$\begin{aligned} & \frac{\mathrm{d}x_1}{\mathrm{d}t}=\widehat{{\alpha }_1\left( w\right) }x^{g_{11}}_1x^{g_{21}}_2-\widehat{{\beta }_1(w)}x_1 \end{aligned}$$

2b $$\begin{aligned} & \frac{\mathrm{d}x_2}{\mathrm{d}t}=\widehat{{\alpha }_2\left( w\right) }x^{g_{12}}_1x^{g_{22}}_2-\widehat{{\beta }_2(w)}x_2 \end{aligned}$$

3 $$\begin{aligned} \frac{dw}{dt}=\mathrm {\upmu }w{\mathrm{log} \left( \sigma \frac{L_w}{w}\right) \ } \end{aligned}$$

where ${x_1=x}_1(t)$ and $x_2=x_2(t)$ are the number of osteoclasts and osteoblasts, respectively; $\widehat{{\alpha }_I\left( w\right) }$ and $\widehat{{\beta }_J(w)}$ represent the activity either cell production or removal, respectively, depending on the tumor (Eq. 3). The tumor is represented by $w=w(t)$. In addition, $g_{ij}$ describes the net effectiveness of osteoclast -or osteoblast- derived autocrine or paracrine factors (Komarova et al. 2003). Also, $\mathrm {\upmu }$ represents the tumor cell proliferation rate, $L_w$ is the upper limit value for the tumor cells, and $\sigma$ is a scaling constant of tumor growth. It is necessary to complement Eq. (2) and (3) with the initial conditions, given by:

4a $$\begin{aligned} & {} x_1(0)=x_{10} \end{aligned}$$

4b $$\begin{aligned}{} & {} x_2(0)=x_{20} \end{aligned}$$

4c $$\begin{aligned}{} & {} w(0)=w_0 \end{aligned}$$

In Eq. (2), the effect of the tumor (*w*) is included in cell production or removal activity. Now, Eq. (3) can be divided by $L_w$, obtaining:

5 $$\begin{aligned} \frac{\mathrm{d}(w/L_w)}{\mathrm{d}t}=\mathrm {\upmu }\left( \frac{w}{L_w}\right) {\mathrm{log} \left( \sigma \frac{L_w}{w}\right) \ } \end{aligned}$$

Using Eq. (5), values for *D* can be defined by $w/L_w$, then Eq. (5) can be written as follows:

6 $$\begin{aligned} \frac{\mathrm{d}D}{\mathrm{d}t}=\mathrm {\upmu }D{\mathrm{log} \left( \frac{\sigma }{D}\right) \ } \end{aligned}$$

On the other hand, $\widehat{{\alpha }_I\left( D\right) }$ and $\widehat{{\beta }_J(w)}$ could have a general structure, for instance $\widehat{{\gamma }_i(w)}$, given by (7):

7 $$\begin{aligned} \widehat{{\gamma }_i\left( w\right) }= {\left\{ \begin{array}{ll} {\gamma }_if(w) &{} \mathrm{multiplicative}\,\mathrm{form} \\ \frac{{\gamma }_i}{f(w)} &{} \mathrm{inverse}\,\mathrm{multiplicative}\,\mathrm{form} \\ {\gamma }_i &{} \mathrm{original}\,\mathrm{form} \end{array}\right. } \end{aligned}$$

Where $f\left( w\right)$ can be chosen in different ways, but here, a “logistic” form was chosen, such that:

8 $$\begin{aligned} f\left( w\right) =k\left( \frac{w_{max}-w}{w_{max}}\right) =k\left( 1-D\right) \end{aligned}$$

Thus, *w* can be replaced with *D* (with $0\le D<1$) in Eq. (2). Therefore, Eqs. (2) and (3) will become (9):

9a $$\begin{aligned}{} & {} \frac{\mathrm{d}x_1}{\mathrm{d}t}=\widehat{{\alpha }_1\left( D\right) }x^{g_{11}}_1x^{g_{21}}_2-\widehat{{\beta }_1(D)}x_1 \end{aligned}$$

9b $$\begin{aligned}{} & {} \frac{\mathrm{d}x_2}{\mathrm{d}t}=\widehat{{\alpha }_2\left( D\right) }x^{g_{12}}_1x^{g_{22}}_2-\widehat{{\beta }_2(D)}x_2 \end{aligned}$$

9c $$\begin{aligned}{} & {} \frac{\mathrm{d}D}{\mathrm{d}t}=\mathrm {\upmu }D{\mathrm{log} \left( \frac{\sigma }{D}\right) \ }\end{aligned}$$

9d $$\begin{aligned}{} & {} \frac{\mathrm{d}z}{\mathrm{d}t}={-k}_1y_1+k_2y_2 \end{aligned}$$

where $\widehat{{\alpha }_I\left( D\right) }$ and $\widehat{{\beta }_J(w)}$ can be studied with different combinations the potential biological characteristics of the effect of the tumor on the bone remodeling process and can be selected from different scenarios provided in Table 1. In Eq. (9d), $z=z(t)$ is the bone mass; $k_i$ is the normalized activity of bone resorption and formation (for $i=1$and $i=2$, respectively); and $y_i=y_i(t,D)$ is the numbers of cells that are resorbing or forming bone. Also, this fourth equation (9d) shows the bone mass change which remains the same as in Komarova et al. (2003) and is expressed as a percentage of the initial mass, according to osteoclasts and osteoblasts numbers, as follows (repeated):

10 $$\begin{aligned} {2y}_i=\left( x_i-\overline{x_i}\right) + \mid \left( x_i-\overline{x_i}\right) \mid \end{aligned}$$

In addition, those ${\overline{x}}_i$ and $\overline{D}$ are the unique nontrivial steady-state numbers of cells and tumor damage percentage, respectively. ${\overline{x}}_i$ and $\overline{D}$ will be obtained in the next section called stability analysis.

**Table 1:** Original model parameters

Scenarios	$\widehat{{\alpha }_1\left( D\right) }$	$\widehat{{\alpha }_2\left( D\right) }$	$\widehat{{\beta }_1\left( D\right) }$	$\widehat{{\beta }_2(D)}$
0	$\alpha _1$	$\alpha _2$	$\beta _1$	$\beta _2$
1	$\frac{\alpha _1}{(1-D)}$	$\alpha _2(1-D)$	$\frac{\beta _1}{(1-D)}$	$\beta _2(1-D)$
2	$\alpha _1(1-D)$	$\frac{\alpha _2}{(1-D)}$	$\beta _1(1-D)$	$\frac{\beta _2}{(1-D)}$
3	$\frac{\alpha _1}{(1-D)}$	$\alpha _2(1-D)$	$\beta _1(1-D)$	$\frac{\beta _2}{(1-D)}$
4	$\alpha _1(1-D)$	$\frac{\alpha _2}{(1-D)}$	$\frac{\beta _1}{(1-D)}$	$\beta _2(1-D)$
5	$\frac{\alpha _1}{(1-D)}$	$\alpha _2$	$\beta _1$	$\beta _2$
6	$\alpha _1$	$\alpha _2$	$\beta _1(1-D)$	$\beta _2$
7	$\alpha _1$	$\frac{\alpha _2}{(1-D)}$	$\beta _1$	$\beta _2$
8	$\alpha _1$	$\alpha _2$	$\beta _1$	$\beta _2(1-D)$

#### Stability analysis

Given Eq. (9a) to (9c), $\frac{dx_1}{dt}=f_{x_1}(x_1,x_2,D)$, $\frac{dx_2}{dt}=f_{x_2}(x_1,x_2,D)$, and $\frac{dD}{dt}=f_{D}(D)$, we can define $\overline{x}_1$, $\overline{x}_2$, $\overline{D}$ which will be the steady states, resulting from the solution of $f_{x_1}(\overline{x}_1,\overline{x}_2,\overline{D})=0$, $f_{x_2}(\overline{x}_1,\overline{x}_2,\overline{D})=0$, and $f_{D}(\overline{D})=0$. Therefore, using Eq. (9), the steady states can be obtained as follows:

11a $$\begin{aligned}{} & {} {\overline{x}}_1={\left( \frac{\widehat{{\beta }_1(\sigma )}}{\widehat{{\alpha }_1\left( \sigma \right) }}\right) }^{(1-g_{22})/\gamma }{\left( \frac{\widehat{{\beta }_2(\sigma )}}{\widehat{{\alpha }_2\left( \sigma \right) }}\right) }^{g_{21}/\gamma } \end{aligned}$$

11b $$\begin{aligned}{} & {} {\overline{x}}_2={\left( \frac{\widehat{{\beta }_1(\sigma )}}{\widehat{{\alpha }_1\left( \sigma \right) }}\right) }^{g_{12}/\gamma }{\left( \frac{\widehat{{\beta }_2(\sigma )}}{\widehat{{\alpha }_2\left( \sigma \right) }}\right) }^{{(1-g}_{11})/\gamma } \end{aligned}$$

11c $$\begin{aligned}{} & {} \overline{D} = \sigma \end{aligned}$$

where $\gamma =g_{12}g_{21}-(1-g_{11})(1-g_{22})$ and $0\le \sigma <1$. In Table 2, there are the steady states for all the scenarios proposed for this model.

**Table 2:** Steady-state solutions in each scenario

Scenarios	Steady states
0, 1, and 2	$$\begin{array}{l} {\overline{x}}_1={\left( \frac{{\beta }_1}{{\alpha }_1}\right) }^{(1-g_{22})/\gamma }{\left( \frac{{\beta }_2}{{\alpha }_2}\right) }^{g_{21}/\gamma } \\ {\overline{x}}_2={\left( \frac{{\beta }_1}{{\alpha }_1}\right) }^{g_{12}/\gamma }{\left( \frac{{\beta }_2}{{\alpha }_2}\right) }^{{(1-g}_{11})/\gamma } \\ \overline{D} = \sigma \end{array}$$
3	$$\begin{array}{l} {\overline{x}}_1={{\left( 1-\sigma \right) }^{\frac{2\left( 1-g_{22}-g_{21}\right) }{\gamma }}\left( \frac{{\beta }_1}{{\alpha }_1}\right) }^{\frac{\left( 1-g_{22}\right) }{\gamma }}{\left( \frac{{\beta }_2}{{\alpha }_2}\right) }^{\frac{g_{21}}{\gamma }} \\ {\overline{x}}_2={\left( 1-\sigma \right) }^{\frac{2\left( -1+g_{11}+g_{12}\right) }{\gamma }}{\left( \frac{{\beta }_1}{{\alpha }_1}\right) }^{\frac{g_{12}}{\gamma }}{\left( \frac{{\beta }_2}{{\alpha }_2}\right) }^{\frac{\left( 1-g_{11}\right) }{\gamma }} \\ \overline{D} = \sigma \end{array}$$
4	$$\begin{array}{l} {\overline{x}}_1={{\left( 1-\sigma \right) }^{\frac{2\left( -1+g_{22}+g_{21}\right) }{\gamma }}\left( \frac{{\beta }_1}{{\alpha }_1}\right) }^{\frac{\left( 1-g_{22}\right) }{\gamma }}{\left( \frac{{\beta }_2}{{\alpha }_2}\right) }^{\frac{g_{21}}{\gamma }} \\ {\overline{x}}_2={\left( 1-\sigma \right) }^{\frac{2\left( 1-g_{11}-g_{12}\right) }{\gamma }}{\left( \frac{{\beta }_1}{{\alpha }_1}\right) }^{\frac{g_{12}}{\gamma }}{\left( \frac{{\beta }_2}{{\alpha }_2}\right) }^{\frac{\left( 1-g_{11}\right) }{\gamma }} \\ \overline{D} = \sigma \end{array}$$
5 and 6	$$\begin{array}{l} {\overline{x}}_1={{\left( 1-\sigma \right) }^{\frac{\left( 1-g_{22}\right) }{\gamma }}\left( \frac{{\beta }_1}{{\alpha }_1}\right) }^{\frac{\left( 1-g_{22}\right) }{\gamma }}{\left( \frac{{\beta }_2}{{\alpha }_2}\right) }^{\frac{g_{21}}{\gamma }} \\ {\overline{x}}_2={\left( 1-\sigma \right) }^{\frac{g_{12}}{\gamma }}{\left( \frac{{\beta }_1}{{\alpha }_1}\right) }^{\frac{g_{12}}{\gamma }}{\left( \frac{{\beta }_2}{{\alpha }_2}\right) }^{\frac{\left( 1-g_{11}\right) }{\gamma }} \\ \overline{D} = \sigma \end{array}$$
7 and 8	$$\begin{array}{l} {\overline{x}}_1={{\left( 1-\sigma \right) }^{\frac{g_{21}}{\gamma }}\left( \frac{{\beta }_1}{{\alpha }_1}\right) }^{\frac{\left( 1-g_{22}\right) }{\gamma }}{\left( \frac{{\beta }_2}{{\alpha }_2}\right) }^{\frac{g_{21}}{\gamma }} \\ {\overline{x}}_2={\left( 1-\sigma \right) }^{\frac{\left( 1-g_{11}\right) }{\gamma }}{\left( \frac{{\beta }_1}{{\alpha }_1}\right) }^{\frac{g_{12}}{\gamma }}{\left( \frac{{\beta }_2}{{\alpha }_2}\right) }^{\frac{\left( 1-g_{11}\right) }{\gamma }} \\ \overline{D} = \sigma \end{array}$$

As shown in Table 2, the steady states can be written, in a general form, as follows:

12a $$\begin{aligned}{} & {} {\overline{x}}_1=h_1(\sigma ){\left( \frac{{\beta }_1}{{\alpha }_1}\right) }^{(1-g_{22})/\gamma }{\left( \frac{{\beta }_2}{{\alpha }_2}\right) }^{g_{21}/\gamma }\end{aligned}$$

12b $$\begin{aligned}{} & {} {\overline{x}}_2={h_2(\sigma )\left( \frac{{\beta }_1}{{\alpha }_1}\right) }^{g_{12}/\gamma }{\left( \frac{{\beta }_2}{{\alpha }_2}\right) }^{{(1-g}_{11})/\gamma } \end{aligned}$$

12c $$\begin{aligned}{} & {} \overline{D} = \sigma \end{aligned}$$

where each $h_i(\sigma )$ can be found using Table 2. Thus, the dynamical system behavior was studied through the approach followed by Murray (2002). The Routh Hurwitz approach is employed to study the system’s stability. Therefore, the Jacobian $\varvec{J}\left( {\overline{x}}_1,{\overline{x}}_2,\overline{D}\right)$, associated with the system of equations Eqs. (9a),(9b), can be defined, which may be obtained by employing the partial derivative of each equation such that $\varvec{J}\left( {\overline{x}}_1,{\overline{x}}_2,\overline{\varvec{D}}\right) ={\left. \frac{\partial f_i}{\partial x_j}\right| }_{\left( {\overline{x}}_1,{\overline{x}}_2,\overline{\varvec{D}}\right) }$. Hence, the entries of the Jacobian are obtained as follows:

13 $$\begin{aligned} \begin{aligned}&J_{11}=g_{11}\widehat{{\alpha }_1\left( \sigma \right) }h^{g_{11}-1}_1\left( \sigma \right) h^{g_{21}}_2\left( \sigma \right) \left( \frac{{\beta }_1}{{\alpha }_1}\right) -\widehat{{\beta }_1\left( \sigma \right) }\\&J_{12}=g_{21}\widehat{{\alpha }_1\left( \sigma \right) }h^{g_{11}}_1\left( \sigma \right) h^{g_{21}-1}_2\left( \sigma \right) {\left( \frac{{\beta }_1}{{\alpha }_1}\right) }^{\frac{g_{11}\left( 1-g_{22}\right) +g_{12}\left( g_{21}-1\right) }{\gamma }}\\&J_{13}={\left. \frac{\partial \widehat{{\alpha }_1\left( D\right) }}{\partial D}\right] }_{D=\sigma }h^{g_{11}}_1\left( \sigma \right) h^{g_{21}}_2\left( \sigma \right) {\left( \frac{{\beta }_1}{{\alpha }_1}\right) }^{\frac{g_{11}\left( 1-g_{22}\right) +g_{12}g_{21}}{\gamma }}{\left( \frac{{\beta }_2}{{\alpha }_2}\right) }^{\frac{g_{21}}{\gamma }}\\&\qquad -{\left. \frac{\partial \widehat{{\beta }_1\left( D\right) }}{\partial D}\right] }_{D=\sigma }h_1(\sigma ){\left( \frac{{\beta }_1}{{\alpha }_1}\right) }^{(1-g_{22})/\gamma }{\left( \frac{{\beta }_2}{{\alpha }_2}\right) }^{g_{21}/\gamma }\\&J_{21}=g_{12}\widehat{{\alpha }_2\left( \sigma \right) }h^{g_{12}-1}_1\left( \sigma \right) h^{g_{22}}_1\left( \sigma \right) {\left( \frac{{\beta }_1}{{\alpha }_1}\right) }^{\frac{g_{22}+g_{12}-1}{\gamma }}{\left( \frac{{\beta }_2}{{\alpha }_2}\right) }^{\frac{{{g_{22}(1-g}_{11})+g}_{21}{(g}_{12}-1)}{\gamma }}\\&J_{22}=g_{22}\widehat{{\alpha }_2\left( \sigma \right) }h^{g_{12}}_1(\sigma )h^{{(g}_{22}-1)}_2(\sigma )\left( \frac{{\beta }_2}{{\alpha }_2}\right) -\widehat{{\beta }_2(\sigma )}\\&J_{31}=0=J_{32}\\&J_{33}=-\frac{\mathrm {\upmu }}{{\mathrm{ln} 10\ }}\\ \end{aligned} \end{aligned}$$

The Jacobian given in (13) has three invariants, which can be written using the determinant and trace of the original two Komarova’s model equations, i.e., employing the minor ($M_{33}$) of $\varvec{J}$ given by $\mathrm {p=}M_{33}=J_{11}J_{22}-J_{12}J_{21}$ and the trace of the submatrix of the first two columns and rows, called $\mathrm{q}=J_{11}+J_{22}$. Furthermore, the characteristic equation will be:

14 $$\begin{aligned} {\lambda }^3-{\lambda }^2\left( \mathrm {p+}J_{33}\right) +\left( J_{33}\mathrm {p+q}\right) \lambda -J_{33}\mathrm {q=0} \end{aligned}$$

Using the notation given in Murray (2002), the invariants of the matrix given by the Jacobian are defined:

15 $$\begin{aligned} \begin{aligned}&a_1=-\left( \mathrm {p+}J_{33}\right) \\&a_2=J_{33}\mathrm {p+q}\\&a_3=-J_{33}\mathrm{q}\\ \end{aligned} \end{aligned}$$

Hence, using the Routh–Hurwitz conditions (Murray 2002), the system has its eigenvalues on the left complex semi-plane, and therefore, the real part of those eigenvalues is negative in order to ensure the stability of the system if the following constraints are fulfilled:

16 $$\begin{aligned} \begin{aligned} a_1>0;\ a_3>0;\ \ a_1a_2-a_3>0 \end{aligned} \end{aligned}$$

Through Eq. (16), it is possible to study the system’s stability and search for parameters that must be used. However, in this work, one of the main purposes is to investigate the effect of tumor growth on the physiologically bone remodeling process. Accordingly, the physiological bone remodeling parameters given in Ryser et al. (2010) and reported in Table 3 were used, such that it is possible to maintain the real negative eigenvalues. Thus, a different behavior of the bone remodeling process in the presence of a tumor may be obtained.

**Table 3:** Parameters used in this work

Parameter	Action	Value
${\mathrm {\upalpha }}_{\mathrm{1}}$	Osteoclast production rate	3 osteoblasts day${}^{-1\ \ }$ (Komarova et al. 2003)
${\mathrm {\upalpha }}_{\mathrm{2}}$	Osteoblast production rate	4 osteoblasts day${}^{-1}$ (Komarova et al. 2003)
${\beta }_1$	Osteoclast removal rate	0.2 osteoclasts day${}^{-1}$ (Komarova et al. 2003)
${\beta }_2$	Osteoblast removal rate	0.02 osteoclasts day${}^{-1\ \ }$ (Komarova et al. 2003)
$g_{11}$	Osteoclast autocrine regulation	1.1 (Komarova et al. 2003)
$g_{21}$	Osteoblast-derived paracrine regulation	− 0.5 (Komarova et al. 2003)
$g_{12}$	Osteoclast-derived paracrine regulation	1 (Komarova et al. 2003)
$g_{22}$	Osteoblast autocrine regulation	0 (Komarova et al. 2003)
$k_1$	Normalized activities of bone resorption	0.093 % osteoclasts${}^{-1}$day${}^{-1}$ (Komarova et al. 2003)
$k_2$	Normalized activities of bone formation	0.0008 % osteoblasts${}^{-1}$day${}^{-1}$ (Komarova et al. 2003)
$\sigma$	Scaling constant of tumor growth	0.05, 0.10, 0.20, 0.40, 0.80, 0.99 dimensionless[used in all the models here presented] (computed)
$\mathrm {\upmu }$	Rate of tumor growth	0.005 day${}^{-1}$ (Ayati et al. 2010)

It is possible to ensure stability in some scenarios using the stability analysis and parameters given in 3. If parameters are fixed using Table 3, it is unnecessary to execute the stability analysis to obtain the range of any parameter. Scenarios that exhibit stability are shown in Table 4. It is important to remark that the stability is given due to the negative real part of the Jacobian’s eigenvalues (13).

**Table 4:** Types of eigenvalues and stability for the first model

Stability type	Scenario (values for $\sigma$, following table 3)							
Type of eigenvalue	1	2	3	4	5	6	7	8
*Stable* $\forall i(Re\left\{ {\lambda }_i\right\} <0)$								
$\exists i(Im\left\{ {\lambda }_i\right\} \ne 0)$		0.05 0.10 0.20 0.40	0.05 0.10 0.20 0.40			0.05 0.10 0.20 0.40 0.80		
$\forall i(Im\left\{ {\lambda }_i\right\} =0)$		0.80 0.99	0.80 0.99			0.99		
*Stable* $\exists i(Re\left\{ {\lambda }_i\right\} =0)$ and one $Re\left\{ {\lambda }_i\right\} <0)$								
$\exists i(Im\left\{ {\lambda }_i\right\} \ne 0)$					0.05 0.10			
*Unstable* $\forall i(Re\left\{ {\lambda }_i\right\} \ge 0)$								
	For all $\sigma$ (Table 3)			For all $\sigma$ (Table 3)	0.20 0.40 0.80 0.99		For all $\sigma$ (Table 3)	For all $\sigma$ (Table 3)

### Tumor with Komarova’s structure

Komarova’s model is revisited, including a new term: the tumor effect on the osteoclasts and osteoblasts rate. Accordingly, the original differential equations are modified, and it is included the tumor rate that also employs two new parameters, named $g_{31}$ and $g_{32}$, as follows:

17a $$\begin{aligned}{} & {} \frac{\mathrm{d}x_1}{\mathrm{d}t}={\mathrm {\upalpha }}_{\mathrm{1}}x^{g_{11}}_1x^{g_{21}}_2D^{g_{31}}-{{\beta }_1x}_1 \end{aligned}$$

17b $$\begin{aligned}{} & {} \frac{\mathrm{d}x_2}{\mathrm{d}t}={\alpha }_2x^{g_{12}}_1x^{g_{22}}_2D^{g_{32}}-{\beta }_2x_2 \end{aligned}$$

17c $$\begin{aligned}{} & {} \frac{\mathrm{d}D}{\mathrm{d}t}=\mathrm {\upmu }D{\mathrm{log} \left( \frac{\sigma }{D}\right) \ } \end{aligned}$$

Hence, one scenario was considered, that can be varied by employing different parameters that should be out of the stability zone in the cancer case.

#### Stability analysis

Similar to the foregoing procedure, it is necessary to understand the stability starting from steady states given by values ${\overline{\varvec{x}}}_{\varvec{1}}$, ${\overline{\varvec{x}}}_{\varvec{2}}$ and $\overline{\varvec{D}}$, which can be obtained using the solution of ${\varvec{f}}_{{\varvec{x}}_{\varvec{1}}}\left( {\overline{\varvec{x}}}_{\varvec{1}},{\overline{\varvec{x}}}_{\varvec{2}},\overline{\varvec{D}}\right) \varvec{=0}$, ${\varvec{f}}_{{\varvec{x}}_{\varvec{2}}}\left( {\overline{\varvec{x}}}_{\varvec{1}},{\overline{\varvec{x}}}_{\varvec{2}},\overline{\varvec{D}}\right) \varvec{=0}$, and ${\varvec{f}}_{\varvec{D}}\left( \overline{\varvec{D}}\right) \varvec{=0}$. Thus, the steady states can be obtained using the following equations :

18a $$\begin{aligned}{} & {} {\overline{\varvec{x}}}_{\varvec{1}}\varvec{=}{\left( \frac{{\boldsymbol{\beta }}_{\varvec{1}}}{{\boldsymbol{\alpha }}_{\varvec{1}}}\right) }^{\varvec{(1-}{\varvec{g}}_{\varvec{22}}\varvec{)/}\boldsymbol{\gamma }}{\left( \frac{{\boldsymbol{\beta }}_{\varvec{2}}}{{\boldsymbol{\alpha }}_{\varvec{2}}}\right) }^{{\varvec{g}}_{\varvec{21}}\varvec{/}\boldsymbol{\gamma }}{\boldsymbol{\sigma }}^{\frac{{\varvec{g}}_{\varvec{31}}\varvec{-}{\varvec{g}}_{\varvec{31}}{\varvec{g}}_{\varvec{22}}\varvec{-}{\varvec{g}}_{\varvec{21}}{\varvec{g}}_{\varvec{32}}}{\boldsymbol{\gamma }}} \end{aligned}$$

18b $$\begin{aligned}{} & {} {\overline{\varvec{x}}}_{\varvec{2}}\varvec{=}{\left( \frac{{\boldsymbol{\beta }}_{\varvec{1}}}{{\boldsymbol{\alpha }}_{\varvec{1}}}\right) }^{{\varvec{g}}_{\varvec{12}}\varvec{/}\boldsymbol{\gamma }}{\left( \frac{{\boldsymbol{\beta }}_{\varvec{2}}}{{\boldsymbol{\alpha }}_{\varvec{2}}}\right) }^{{\varvec{(1-}\varvec{g}}_{\varvec{11}}\varvec{)/}\boldsymbol{\gamma }}{\boldsymbol{\sigma }}^{\frac{{\varvec{g}}_{\varvec{32}}{\varvec{g}}_{\varvec{11}}\varvec{-}{\varvec{g}}_{\varvec{31}}{\varvec{g}}_{\varvec{12}}\varvec{-}{\varvec{g}}_{\varvec{32}}}{\boldsymbol{\gamma }}} \end{aligned}$$

18c $$\begin{aligned}{} & {} \overline{\varvec{D}}\varvec{ = }\boldsymbol{\sigma } \end{aligned}$$

Employing (18), it is possible to write those equations as follows:

19a $$\begin{aligned}{} & {} {\overline{x}}_1=m_1(\sigma ){\left( \frac{{\beta }_1}{{\alpha }_1}\right) }^{(1-g_{22})/\gamma }{\left( \frac{{\beta }_2}{{\alpha }_2}\right) }^{g_{21}/\gamma } \end{aligned}$$

19b $$\begin{aligned}{} & {} {\overline{x}}_2={m_2(\sigma )\left( \frac{{\beta }_1}{{\alpha }_1}\right) }^{g_{12}/\gamma }{\left( \frac{{\beta }_2}{{\alpha }_2}\right) }^{{(1-g}_{11})/\gamma }\end{aligned}$$

19c $$\begin{aligned}{} & {} \overline{D}\varvec{ = }\boldsymbol{\sigma } \end{aligned}$$

Additionally, the Jacobian defined $\varvec{J}\left( {\overline{x}}_1,{\overline{x}}_2,\overline{\varvec{D}}\right)$ can be associated with (17a) to (17c), which may be obtained by employing the partial derivative of each equation such that $\varvec{J}\left( {\overline{x}}_1,{\overline{x}}_2,\overline{\varvec{D}}\right) ={\left. \frac{\partial f_i}{\partial x_j}\right| }_{\left( {\overline{x}}_1,{\overline{x}}_2,\overline{\varvec{D}}\right) }$, obtaining the entries of the Jacobian as follows:

20 $$\begin{aligned} \begin{aligned}&J_{11}=g_{11}{\alpha }_1m^{g_{11}-1}_1\left( \sigma \right) m^{g_{21}}_2\left( \sigma \right) \left( \frac{{\beta }_1}{{\alpha }_1}\right) {\sigma }^{g_{31}}-{\beta }_1 \\&J_{12}=g_{21}{\alpha }_1m^{g_{11}}_1\left( \sigma \right) m^{g_{21}-1}_2\left( \sigma \right) {\left( \frac{{\beta }_1}{{\alpha }_1}\right) }^{\frac{g_{11}\left( 1-g_{22}\right) +g_{12}\left( g_{21}-1\right) }{\gamma }}{\left( \frac{{\beta }_2}{{\alpha }_2}\right) }^{\frac{g_{11}+g_{21}-1}{\gamma }}{\sigma }^{g_{31}}\\&J_{13}={\alpha }_1g_{31}m^{g_{11}}_1\left( \sigma \right) m^{g_{21}}_2\left( \sigma \right) {\left( \frac{{\beta }_1}{{\alpha }_1}\right) }^{\frac{g_{11}\left( 1-g_{22}\right) +g_{12}g_{21}}{\gamma }}{\left( \frac{{\beta }_2}{{\alpha }_2}\right) }^{\frac{g_{21}}{\gamma }}{\sigma }^{g_{31}-1}\\&J_{21}=g_{12}\widehat{{\alpha }_2\left( \sigma \right) }h^{g_{12}-1}_1\left( \sigma \right) h^{g_{22}}_1\left( \sigma \right) {\left( \frac{{\beta }_1}{{\alpha }_1}\right) }^{\frac{g_{22}+g_{12}-1}{\gamma }}{\left( \frac{{\beta }_2}{{\alpha }_2}\right) }^{\frac{{{g_{22}(1-g}_{11})+g}_{21}{(g}_{12}-1)}{\gamma }}\\&J_{22}=g_{22}{\alpha }_2m^{g_{12}}_1(\sigma )m^{{(g}_{22}-1)}_2(\sigma )\left( \frac{{\beta }_2}{{\alpha }_2}\right) {\sigma }^{g_{32}}-{\beta }_2\\&J_{23}=g_{22}{\alpha }_2m^{g_{12}}_1(\sigma )m^{{(g}_{22}-1)}_2(\sigma )\left( \frac{{\beta }_2}{{\alpha }_2}\right) {\sigma }^{g_{32}}-{\beta }_2\\&J_{31}=0=J_{32}\\&J_{33}=-\frac{\mathrm {\upmu }}{{\mathrm{ln} 10\ }}\\ \end{aligned} \end{aligned}$$

**Fig. 2:**
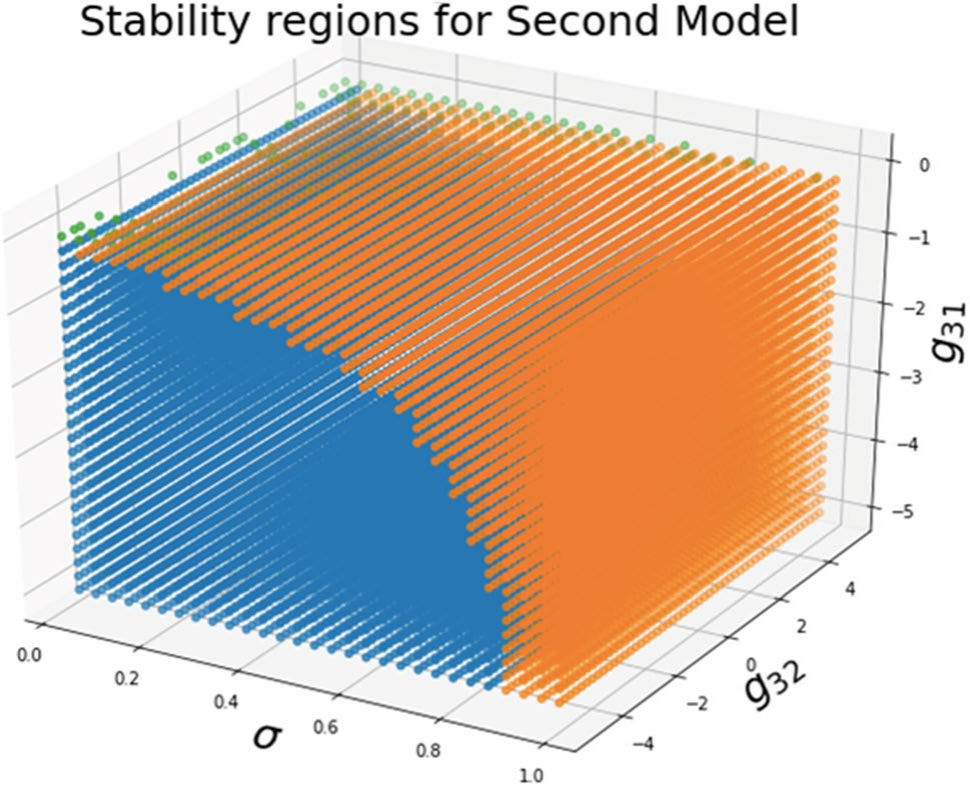
Stability regions for the second model: blue: null imaginary part, all real part negative; orange: one root real negative and two complex with real negative part; green: one root real negative and two pure imaginary

Equations (14), (15), and (16), and Table 1 were used for conducting a stability analysis in pursuit of g31 and g32. In contrast, it is necessary to obtain the values of sigma, g31, and g32 and the zone of stability with the first model, as it is shown in Fig. 2.

### Tumor affecting the Komarova’s structure through the paracrine and autocrine parameters

Ayati et al. (2010) proposed a mathematical approach based on Komarova’s model. In that model, the paracrine and autocrine effects change depending on the tumor cell density. Also, the equation for tumor cells is based on a logarithmic law given by (21c). The model that they proposed was also implemented in two dimensions. Here, a similar model will be proved with two possibilities, (1) the model for cancer cells used in Ayati et al. (2010) and (2) the structure used in this paper. The equations implemented are (21):

21a $$\begin{aligned}{} & {} \frac{\mathrm{d}x_1}{\mathrm{d}t}={\mathrm {\upalpha }}_{\mathrm{1}}x^{\widetilde{g_{11}}}_1x^{\widetilde{g_{21}}}_2-{{\beta }_1x}_1 \end{aligned}$$

21b $$\begin{aligned}{} & {} \frac{\mathrm{d}x_2}{\mathrm{d}t}={\alpha }_2x^{\widetilde{g_{12}}}_1x^{\widetilde{g_{22}}}_2-{\beta }_2x_2 \end{aligned}$$

21c $$\begin{aligned}{} & {} \frac{\mathrm{d}D}{\mathrm{d}t}=\mathrm {\upmu }D{\mathrm{log} \left( \frac{\sigma }{D}\right) \ } \end{aligned}$$

where $\widetilde{g_{11}}(D)=g_{11}\left( 1+r_{11}\frac{D}{\sigma }\right)$, $\widetilde{g_{21}}(D)=g_{21}\left( 1+r_{21}\frac{D}{\sigma }\right)$, $\widetilde{g_{12}}(D)=g_{12}/\left( 1+r_{12}\frac{D}{\sigma }\right)$, $\widetilde{g_{22}}(D)=g_{22}-r_{22}\frac{D}{\sigma }$, with $r_{ij}$ as constants. There are four new parameters compared to the original Komarova’s model. In Eq. (21), it is possible to calculate the difference from previous equations because values for $g_{ij}$ have been changed conveniently to reproduce cancer’s effects on the biochemical environment of the bone remodeling process. In fact, the term $r_{ij}\frac{D}{\sigma }$ produces the coupling between tumor and osteoclasts/osteoblasts interactions. A complete study of the stability of that model is presented in Ayati et al. (2010), and in this paper it is repeated employing a new methodology.

#### Stability analysis

The stability analysis uses the steady states given by ${\overline{\varvec{x}}}_{\varvec{1}}$, ${\overline{\varvec{x}}}_{\varvec{2}}$ and $\overline{\varvec{D}}$, which can be obtained by using the solution of ${\varvec{f}}_{{\varvec{x}}_{\varvec{1}}}\left( {\overline{\varvec{x}}}_{\varvec{1}},{\overline{\varvec{x}}}_{\varvec{2}},\overline{\varvec{D}}\right) \varvec{=0}$, ${\varvec{f}}_{{\varvec{x}}_{\varvec{2}}}\left( {\overline{\varvec{x}}}_{\varvec{1}},{\overline{\varvec{x}}}_{\varvec{2}},\overline{\varvec{D}}\right) \varvec{=0}$, and ${\varvec{f}}_{\varvec{D}}\left( \overline{\varvec{D}}\right) \varvec{=0}$. Using Eq. (18), the steady states are obtained as follows:

22a $$\begin{aligned} & {\overline{\varvec{x}}}_{\varvec{1}}\varvec{=}{\left( \frac{{\boldsymbol{\beta }}_{\varvec{1}}}{{\boldsymbol{\alpha }}_{\varvec{1}}}\right) }^{\frac{\varvec{1-}{\varvec{g}}_{\varvec{22}}\varvec{+}{\varvec{r}}_{\varvec{22}}}{\varvec{{\Lambda }}}}{\left( \frac{{\boldsymbol{\beta }}_{\varvec{2}}}{{\boldsymbol{\alpha }}_{\varvec{2}}}\right) }^{\frac{{\varvec{g}}_{\varvec{21}}\varvec{(1+}{\varvec{r}}_{\varvec{21}}\varvec{)}}{\varvec{{\Lambda }}}} \end{aligned}$$

22b $$\begin{aligned}{} & {} {\overline{\varvec{x}}}_{\varvec{2}}\varvec{=}{\left( \frac{{\boldsymbol{\beta }}_{\varvec{1}}}{{\boldsymbol{\alpha }}_{\varvec{1}}}\right) }^{\frac{{\varvec{g}}_{\varvec{12}}}{\boldsymbol{\Lambda}\varvec{(1+}{\varvec{r}}_{\varvec{12}}\varvec{)}}}{\left( \frac{{\boldsymbol{\beta }}_{\varvec{2}}}{{\boldsymbol{\alpha }}_{\varvec{2}}}\right) }^{\frac{\varvec{1-}{\varvec{g}}_{\varvec{11}}\varvec{(1+}{\varvec{r}}_{\varvec{11}}\varvec{)}}{\boldsymbol{\Lambda}}} \end{aligned}$$

22c $$\begin{aligned}{} & {} \overline{\varvec{D}}\varvec{ = }\boldsymbol{\sigma } \end{aligned}$$

where $\mathrm {\Lambda } = {(g}_{12}\mathrm {\ /(}\mathrm{1}\mathrm {+}r_{12}\mathrm {))(}\ g_{21}\mathrm {(}\mathrm{1}\mathrm {+}r_{21}\mathrm {))-(}\mathrm{1}\mathrm {-}g_{11}\mathrm {(}\mathrm{1}\mathrm {+}r_{11}\mathrm {))(}\mathrm{1}\mathrm {-}g_{22}\mathrm {+}r_{22}\mathrm {)}$. Using the previously indicated methodology, the Jacobian can be obtained with the entries given by:

23 $$\begin{aligned} \begin{aligned}&J_{11}=\widetilde{g_{11}}(\sigma ){\alpha }_1{\overline{x}}^{\widetilde{g_{11}}\left( \sigma \right) -1}_1{\overline{x}}^{\widetilde{g_{21}}(\sigma )}_2-{\beta }_1 \\&J_{12}=\widetilde{g_{21}}(\sigma ){\alpha }_1{\overline{x}}^{\widetilde{g_{11}}\left( \sigma \right) }_1{\overline{x}}^{\widetilde{g_{21}}\left( \sigma \right) -1}_2\\&J_{13}={\alpha }_1{\overline{x}}^{\widetilde{g_{11}}\left( \sigma \right) }_1{\overline{x}}^{\widetilde{g_{21}}\left( \sigma \right) }_2\left( {\mathrm{ln} \left( {\overline{\varvec{x}}}_{\varvec{1}}\right) \ }{\left. \frac{\partial \widetilde{g_{11}}\left( D\right) }{\partial D}\right| }_{D=\sigma }+\mathrm{ln}({\overline{\varvec{x}}}_{\varvec{2}}){\left. \frac{\partial \widetilde{g_{21}}\left( D\right) }{\partial D}\right| }_{D=\sigma }\right) \\&J_{21}=\widetilde{g_{12}}(\sigma ){\alpha }_2{\overline{x}}^{\widetilde{g_{12}}\left( \sigma \right) -1}_1{\overline{x}}^{\widetilde{g_{22}}(\sigma )}_2\\&J_{22}=\widetilde{g_{22}}(\sigma ){\alpha }_2{\overline{x}}^{\widetilde{g_{12}}\left( \sigma \right) }_1{\overline{x}}^{\widetilde{g_{22}}\left( \sigma \right) -1}_2-{\beta }_2\\&J_{23}={\alpha }_2{\overline{x}}^{\widetilde{g_{12}}\left( \sigma \right) }_1{\overline{x}}^{\widetilde{g_{22}}\left( \sigma \right) }_2\left( {\mathrm{ln} \left( {\overline{\varvec{x}}}_{\varvec{1}}\right) \ }{\left. \frac{\partial \widetilde{g_{12}}\left( D\right) }{\partial D}\right| }_{D=\sigma }+\mathrm{ln}{}({\overline{\varvec{x}}}_{\varvec{2}}){\left. \frac{\partial \widetilde{g_{22}}\left( D\right) }{\partial D}\right| }_{D=\sigma }\right) \\&J_{31}=0=J_{32}\\&J_{33}=-\frac{\mathrm {\upmu }}{{\mathrm{ln} 10\ }}\\ \end{aligned} \end{aligned}$$

**Fig. 3:**
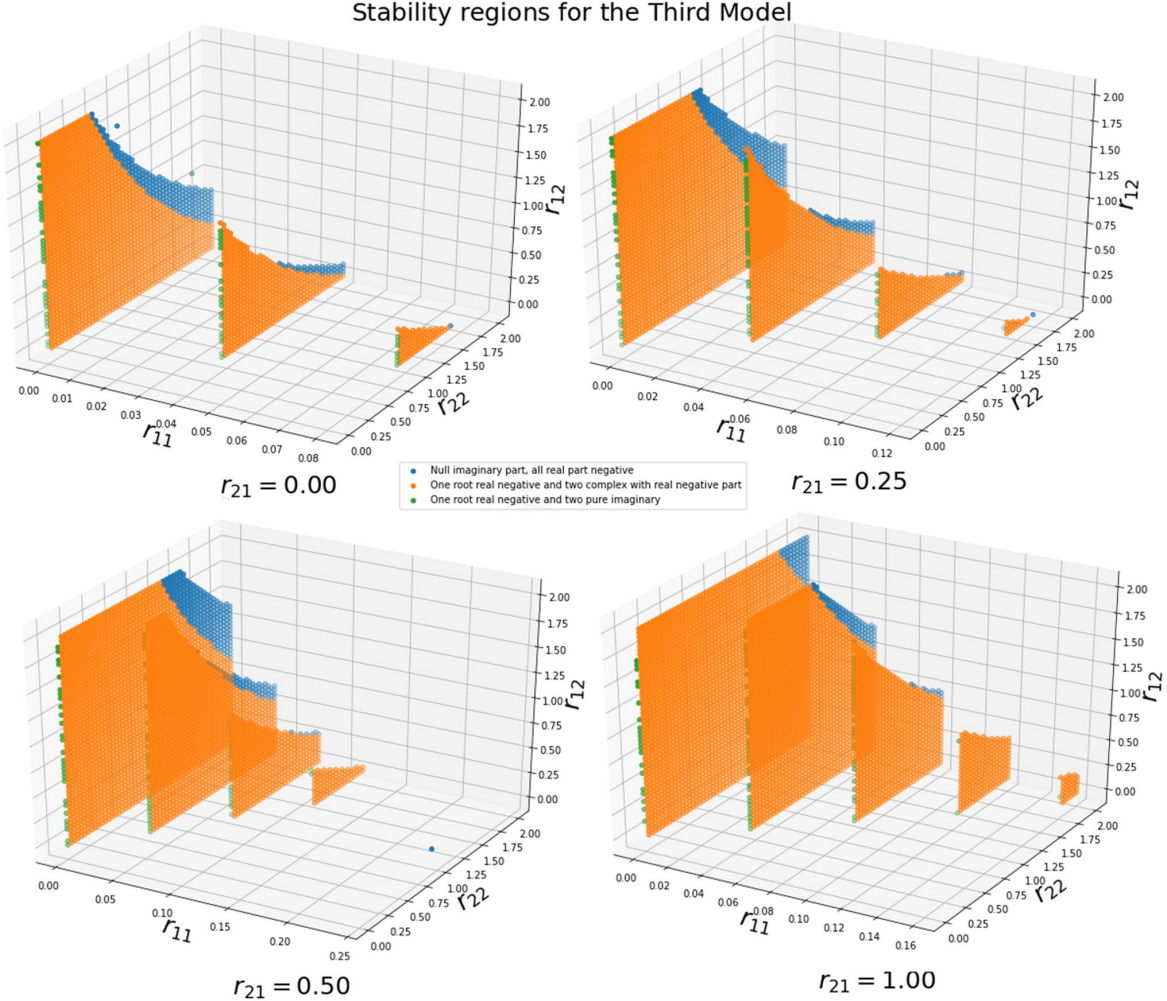
Stability surfaces for parameters $r_{11}$, $r_{12}$, $r_{31}$ and $r_{22}$ in model 3

Equations (21a), (21b), and (21c), and Table 3 were used for running a stability analysis searching $r_{11}$, $r_{12}$, $r_{31}$ and $r_{22}$. By contrast four parameters were obtained using the second model, which require a hypersurface that cannot be represented here, because of that limitation. In Fig. 3, the surfaces for some values of $r_{21}$ are represented.

## Results and discussion

**Fig. 4:**
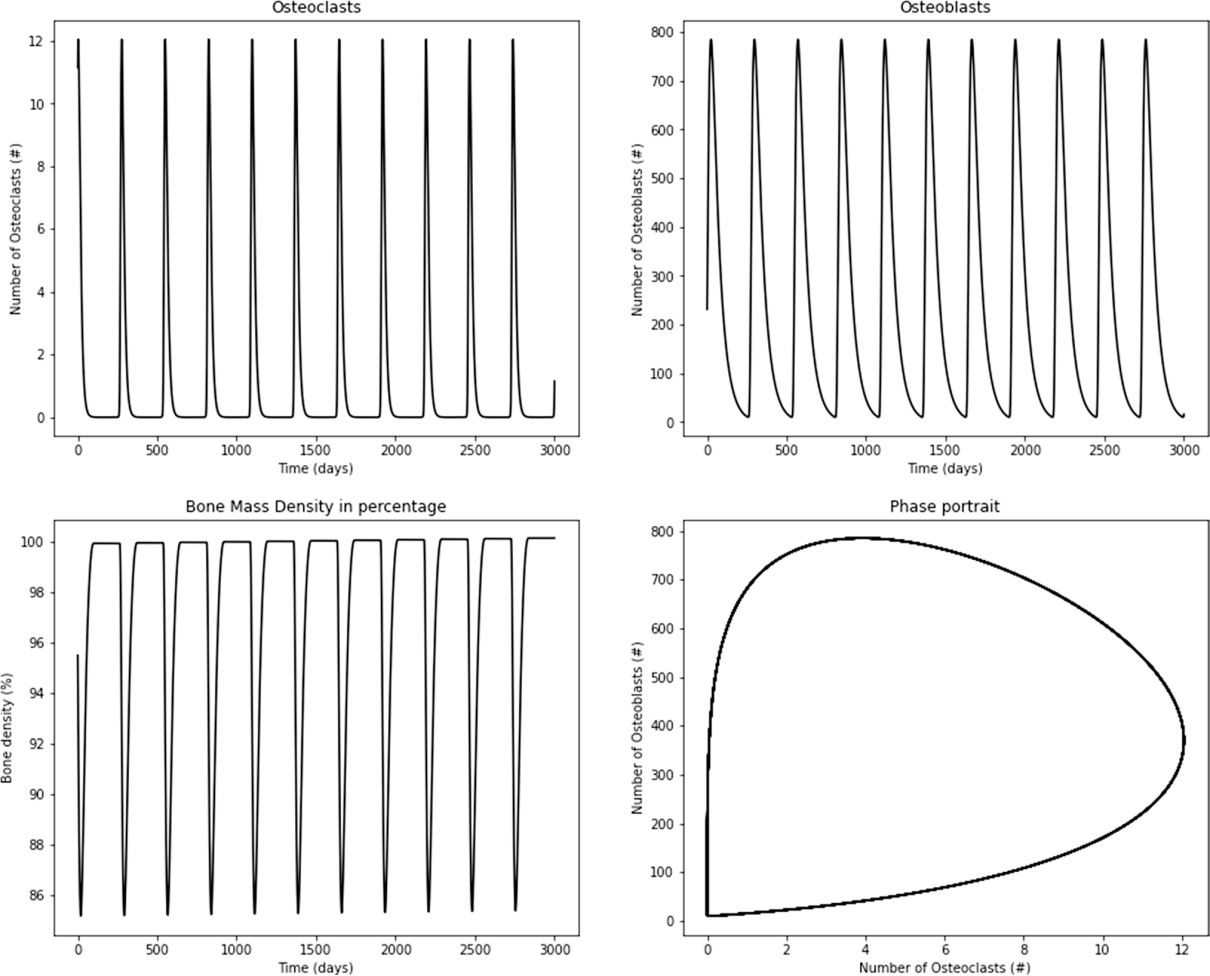
Results for the original bone remodeling process using the Komarova’s model under the parameters given in Table 3

All simulations were performed in Python language with Google $Colab-Team$ compiler version 1.0.1 (Perron and Furnon, Or-tools).The effects of tumor growth on the physiological bone remodeling process were modeled. In the first model proposed and analyzed in this paper, the tumor’s influence on the osteoclasts and osteoblasts was studied throughout terms associated with damage. A second model employed a multiplicative term of tumor density in Komarova’s original model. Finally, those models were compared with Ayati’s one (Ayati et al. 2010). The tumor’s influence on the paracrine and autocrine signaling was included. In the first model, the tumor influence on the bone remodeling process was included as a function of $\alpha$ and $\beta$, i.e., proliferation and removal terms, respectively. Therefore, $\alpha$ and $\beta$ were conveniently changed from a constant value (from Komarova’s original model) to a new function that depends on the tumor cell density and can reproduce effects such as over-proliferation or reduction in bone mass. The removal of bone mass is observed due to a high proliferation of osteoclasts or unbalanced activity of the BMU, similar to metastasis or multiple myeloma (Brigle 2017). Similarly, it is possible to have an uncontrolled increase in abnormal bone mass, like osteoblastoma (Bokhari 2012). The models presented used the underlying Komarova’s model due to its versatility for reproducing bone remodeling processes and its simplicity of its concept behind the mathematics. Figure 4 shows the osteoclast and osteoblast graphs, the bone mass process, and the phase portrait of osteoclasts, representing the limit cycle under the parameters considered in Table 3. This model corresponds to scenario 0, which in Table 1 corresponds to the first model presented. In Fig. 4, the physiological bone remodeling process shows a limit cycle with a repetitive and stable process with a perfect balance between osteoblasts and osteoclasts, producing a continuous removal and construction of the bone mass. The normal bone mass presents periodic solutions with physiological oscillation. Moreover, osteoblasts and osteoclasts have the same periodicity with a maximum amplitude of approximately 800 and 12 cells, respectively. Bone increases and decreases in mass due to the net effect of formation and resorption given by Eq. 9. It is important to mention that the nontrivial steady state (under parameters of Table 3) is provided by 1.1586 and 231.7238 for osteoclasts and osteoblasts, respectively. Additionally, the eigenvalues do not exhibit a real part ($\mathfrak {Re}\{0\}=0$); thus, regular periodic cycles are obtained, as shown in Fig. 4. Complete stability analysis of Komarova’s model can be found at Komarova et al. (2003). All models simulated here used $1.1586 + 10.0000$ and 231.7238 for osteoclasts and osteoblasts, respectively, as initial conditions.

**Fig. 5:**
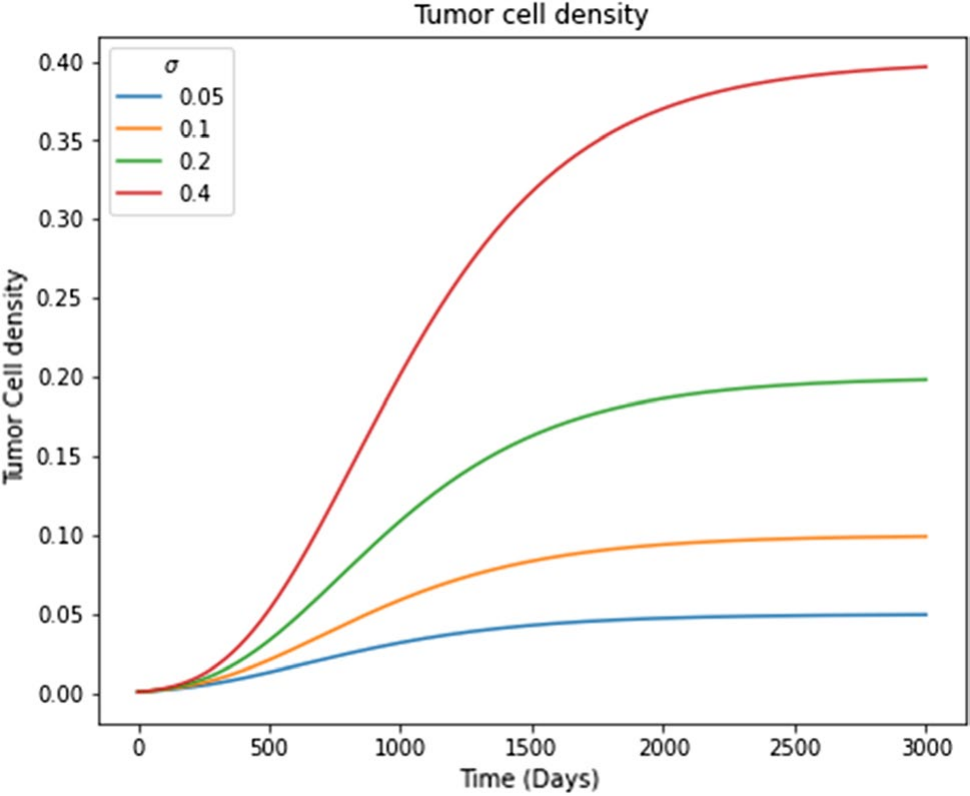
Results for the tumor cell evolution for different values of $\sigma$

Figure 5 shows the tumor evolution through time, corresponding to Eq. 9 for different values of $\sigma$. Here it is possible to observe how $\sigma$ represents the maximum value at which the system is stabilized. Employing this model, similar to that used by Brigle (2017), it is possible to study the influence of tumor growth on the bone remodeling process represented by Komarova’s model.

Using the three models, it is possible to simulate the influence on tumor cell’s growth during bone remodeling process. Although a plethora of examples and outcomes may be produced depending on the type of eigenvalues, in this document just a few were considered.

### Tumor affecting the activity of cell production or removal

This model considers the effect of rewriting terms $\alpha$ (rate of proliferation) and $\beta$ (rate of removal) from constant values to a new deal that depends on the tumor growth called *D*. $\alpha (D)$ and $\beta (D)$ can increase if the new function is the multiplication of the constant previous value times $\frac{1}{1-D}$, or decrease if the constants multiply the factor $1-D$. The value of *D* will be in the range of 0 to 1; therefore, the resulting scenarios combine the constant times, depending on the value of *D*. It is not necessary to include additional autocrine or paracrine parameters, as in models 2 and 3 these factors were included.

**Fig. 6:**
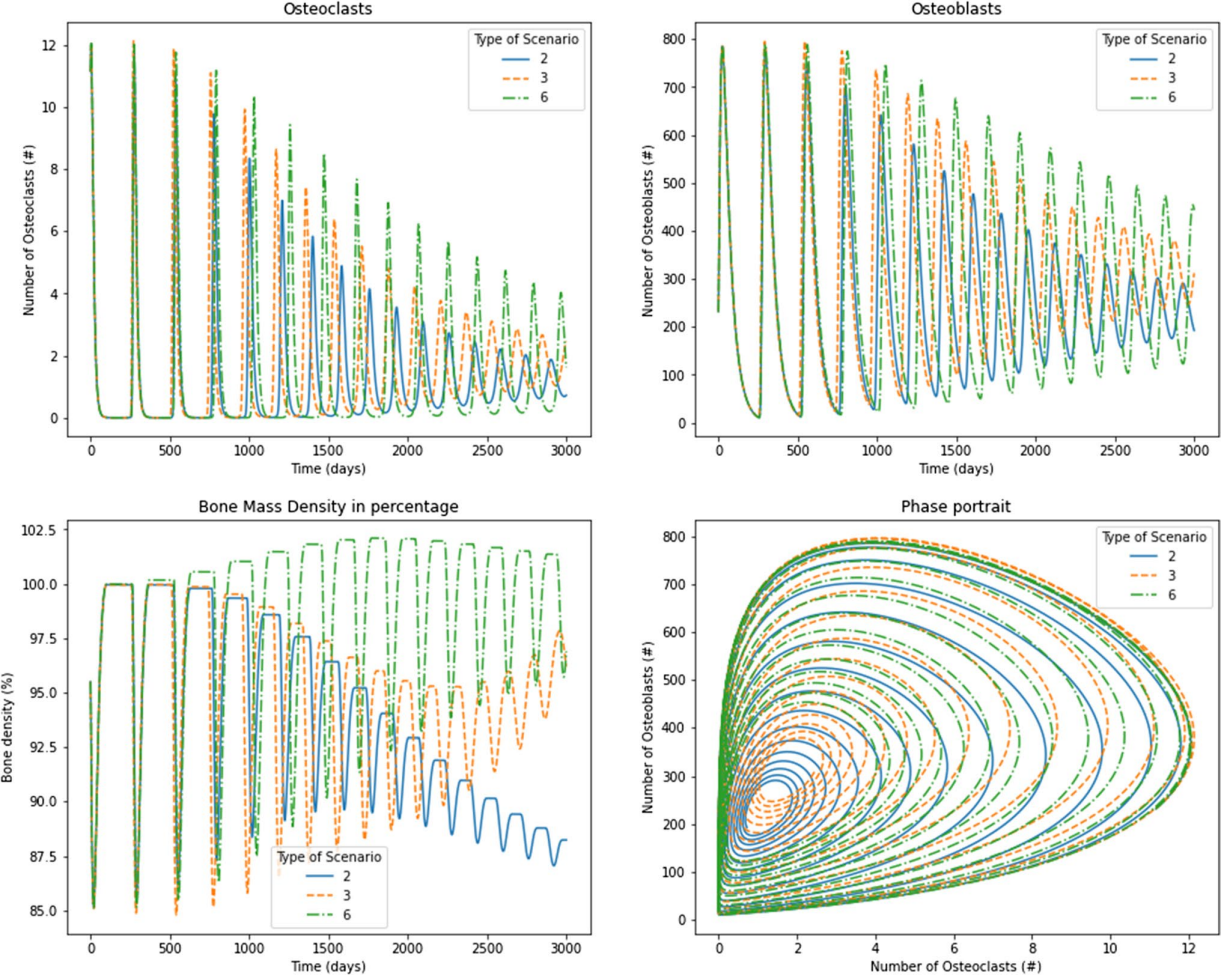
Results for osteoclasts, osteoblasts, bone mass, and the phase portrait for scenarios 2, 3, and 6 under $\sigma =0.05$

**Fig. 7:**
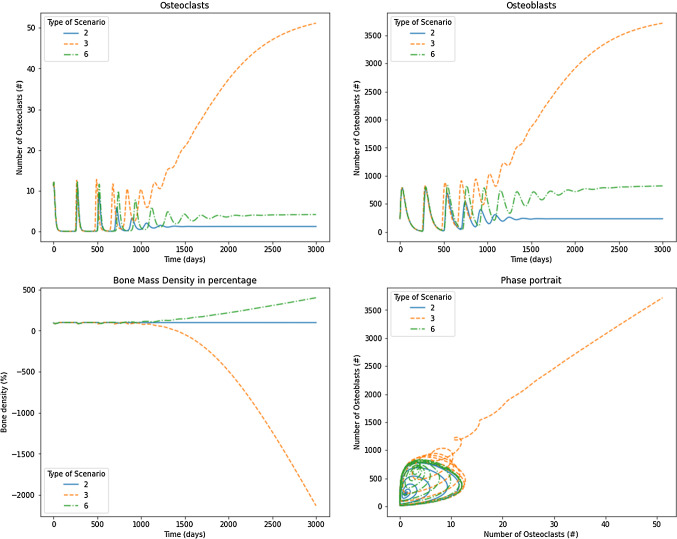
Results for osteoclasts, osteoblasts, bone mass, and the phase portrait for scenarios 2, 3, and 6 under $\sigma =0.40$

Regarding the results consigned in Table 4, the stability analysis showed that the tumor has different effects depending on the functions $\alpha (D)$ and $\beta (D)$. Scenarios 2, 3, and 6 exhibited damped oscillations of osteoblasts and osteoclasts that converge to the nontrivial steady states. Scenario 2 has terms $\alpha _1(1-D)$ and $\beta _1(1-D)$ for osteoclasts production and removal, respectively. The production and the removal are decreased because the tumor grows over time. On the other hand, it produced increased osteoblasts production and removal values. These results correspond to those found in tumors installed into the bone that produces a short remotion of damaged cells (throughout the osteoclasts) and produce excessive new osteoblasts. The net bone mass apposition is decreasing, behavior that can be observed in some bone diseases, such as osteoarthrosis, where excess osteoblasts can produce chondrocytes apoptosis (Song et al. 2021). Similarly, some tumors, such as multiple myeloma, can reduce bone mass due to a decreasing of the formation of bone cells (Joshua et al. 2019). Results showed a decrease in the bone remodeling period for the osteoclasts and osteoblasts, similar to those of Ayati et al. (2010). However, the solution converged to the nontrivial steady state, independent of $\sigma$ as noted in 2, given by 1.1586 and 231.7238 for osteoclasts and osteoblasts, respectively. This convergence corresponds to the negative real eigenvalues found in the stability analysis that promotes damped convergent oscillations compared to the original Komarova’s model, which oscillates regularly in a limit cycle. Although the steady state is independent of $\sigma$, it is possible to observe the effect when $\sigma = 0.05$ is used (Fig. 6), in comparison with $\sigma =0.4$ (Fig. 7) where the convergence is achieved faster. Therefore, the bone mass was decremented substantially until $87\%$ through values $k_1$ and $k_2$ used in Eq. 9 and taken from Table 3.

Scenario 3 has terms $\frac{\alpha _1}{1-D}$ and $\beta _1(1-D)$ for osteoclasts production and removal, respectively. The production is increased, and the removal is decreased because tumor grows over time. On their part, $\alpha _2(1-D)$ and $\frac{\beta _2}{1-D}$ produce declining values of osteoblasts production and an increasing removal of osteoblasts. Although eigenvalues are negative and they have an imaginary part, the phase portrait in Figs. 6 and 7 shows the location of the pair osteoclast, osteoblasts in a region far from the biological sense. In addition, the temporal evolution of osteoclasts and osteoblasts graphs shows a convergence trend over time. In Fig. 7, results showed a temporary decrease in bone mass followed by a slight growth of the mass. The solution for osteoclasts and osteoblasts converged to the nontrivial steady state, depending on $\sigma =0.05$ as noted in Table 2, given by 1.7022 and 307.2492, respectively. By contrast, for $\sigma =0.4$ (Fig. 7), the convergence was achieved faster, reaching a steady state of 53.4325 and 3847.1469 osteoclasts and osteoblasts, respectively, which does not have sense in the biological framework evaluated. However, it is possible to interpret that the results did not have sense after 1000 days when bone mass starts showing negative values. In other words, this scenario shows how $\sigma$ affects the bone remodeling process; as greater is as faster the failure in bone mass exchange is produced. This result is in line with metastasis treatments, in which the bone remodeling cycle and bone mass apposition are promoted through bisphosphonates to avoid patient morbidity (Sindhi and Erdek 2019).

Scenario 6 uses the original Komarova’s model with one change in parameter $\beta _1(1-D)$, which reduces the removal of osteoclast of the system. Figures 6 and 7 show the convergence of osteoclasts and osteoblasts to values that depend on $\sigma$, and in both cases, bone mass apposition grows through time. Figure 6 shows how osteoclasts and osteoblasts (for $\sigma =0.05$) reach the steady state of 1.3171 and 263.4278, respectively. In comparison, Fig. 7, for $\sigma =0.40$ exhibits values for those cells of 4.1549 and 830.9837. The increasing bone mass represents the unbalanced remodeling process which ponderates the osteoblast functioning, similar to metaplasia (Villegas et al. 2009) or carcinomas (Koyama et al. 2021).

**Fig. 8:**
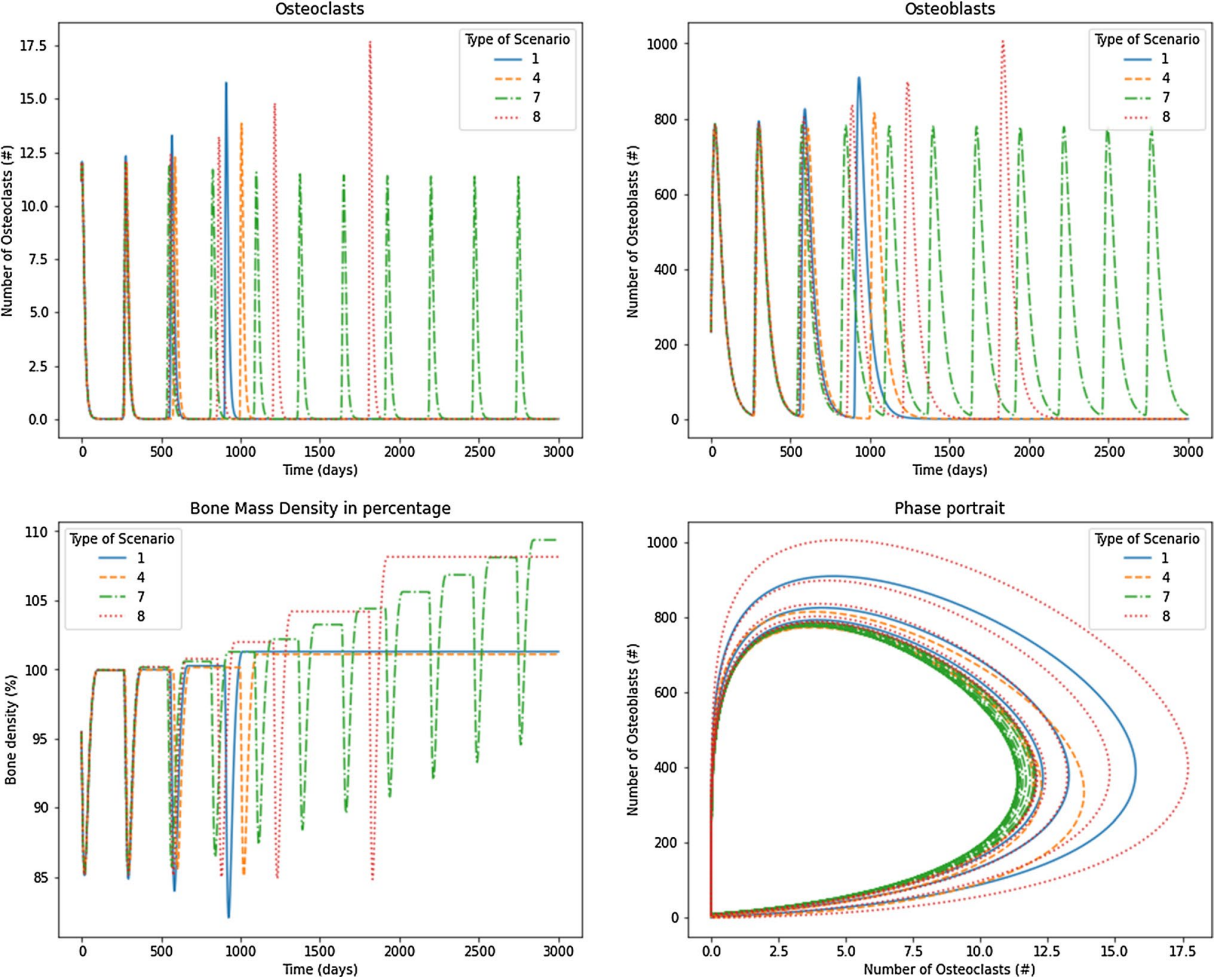
Results for osteoclasts, osteoblasts, bone mass, and the phase portrait for scenarios 1, 4, 7 and 8 under $\sigma =0.05$

**Fig. 9:**
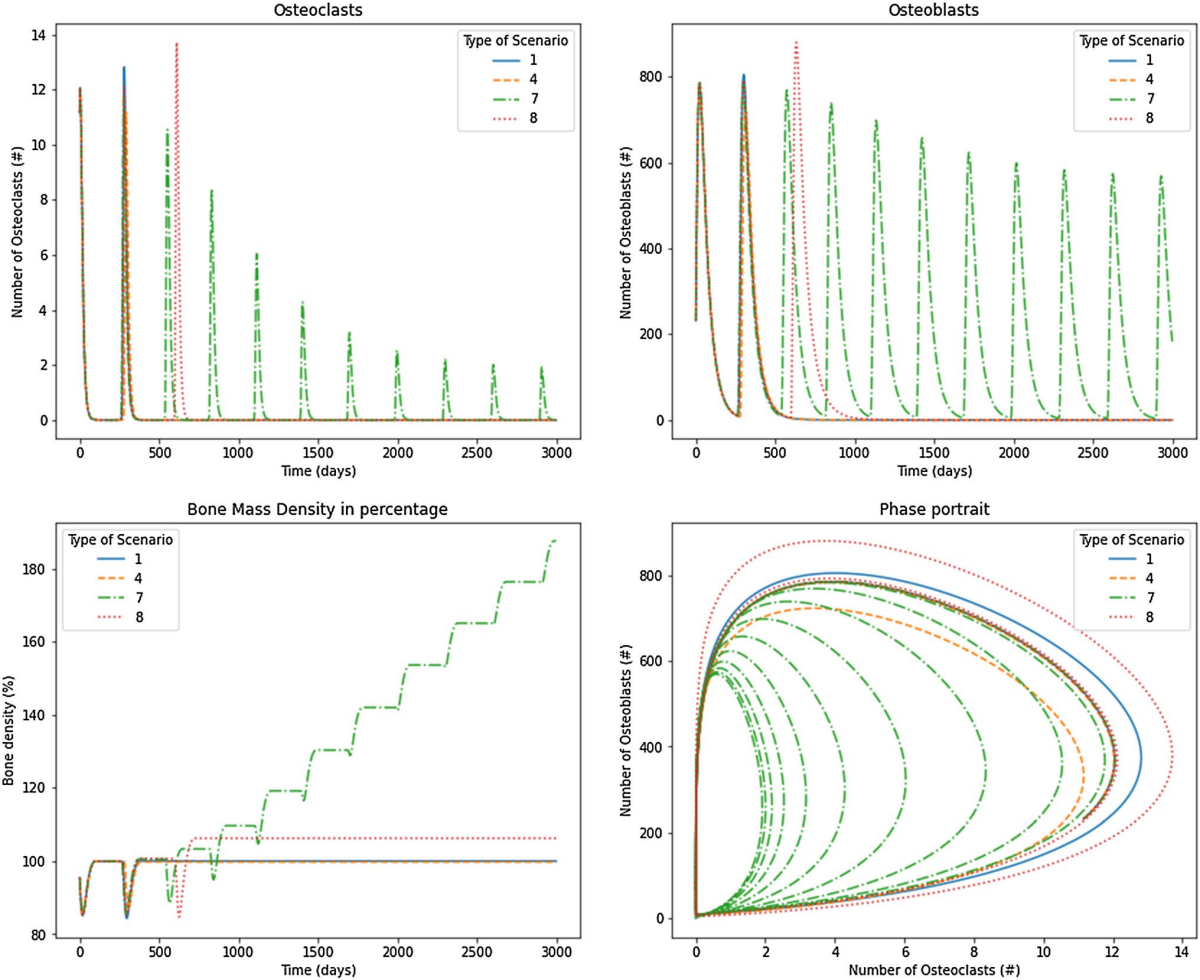
Results for osteoclasts, osteoblasts, bone mass, and the phase portrait for scenarios 1, 4, 7 and 8 under $\sigma =0.80$

Scenarios 1, 4, 7, and 8 are shown in Fig. 8 for $\sigma =0.05$. In particular, these scenarios exhibit one pure negative real eigenvalue and two other eigenvalues with a real positive part, except scenario 7, which has a limit cycle due to the existence of two pure imaginary eigenvalues. In scenario 1, shown in the continued blue line, the nontrivial steady state (which does not depend on the $\sigma$ value) is given by 1.1586 and 231.7238 for osteoclasts and osteoblasts, respectively. The response oscillates, increasing the number of BMU cells, then, the oscillation is lost, and the system is attracted to a value of zero for osteoclasts and osteoblasts. Bone mass density achieved a value of $102\%$ and did not oscillate over time. This behavior can be compared to some diseases that do not correspond to tumor or cancer cases. For instance, Legg-Calve-Perthes disease is common in children (Richard Bowen et al. 1984). This disease is characterized by the reduction in blood supply in the bone, producing a lack of bone precursors cells; meanwhile, the tissue is maintained and not renewed. Because the bone is constantly under mechanical loads, the bone accumulates fractures producing a mechanical collapse. Figure 9 showed that, similar to scenario 1, scenarios 4 and 8 exhibit oscillation lost over time. However, the difference is that those oscillate one extra cycle, and the period of the cycle grows along the time. The nontrivial steady state (it does depend on the $\sigma$ value, following Table 4) is given by 0.7886 and 174.7634 (scenario 4) and by 1.0866 and 228.7713 for osteoclasts and osteoblasts, respectively. The final value of the BMU cell numbers is null. Scenario 7 exhibits a limit cycle with a nontrivial steady state given by $x_1=1.0866$ and $x_2=228.7713$ (depending of $\sigma$). It shows a limit cycle similar to Komarova’s original model with reduced steady-state values compared to the physiological model. Consequently, the osteoclasts’ maximum value is reduced, producing an unbalanced process that augments bone mass construction. This case can represent the process of bone growth and fracture repair after the endochondral stage (Gerber and Ferrara 2000) (Fig. 8).

Figure 9 shows the scenarios 1, 4, 7, and 8 for $\sigma =0.80$. Increasing $\sigma$ from 0.05 to 0.80 produced, in Scenarios 1, 4 and 8, a decreased number of cycles for BMU cells. By contrast, the scenario 7 exhibits a damped oscillation due to one negative eigenvalue, although the other two eigenvalues have pure imaginary ones. The unbalanced osteoclast-osteoblasts system produced an uncontrolled increase in bone mass.

**Fig. 10:**
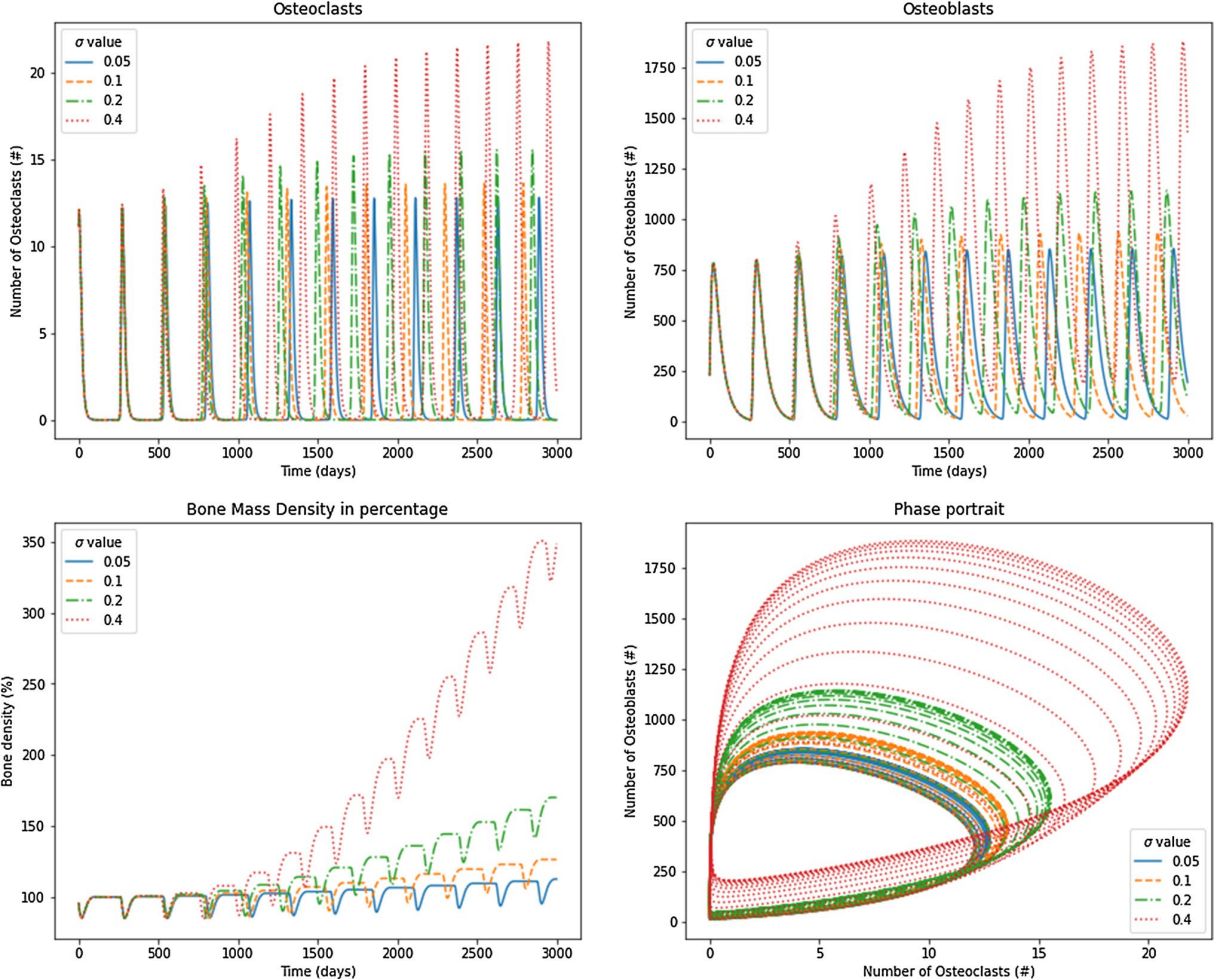
Results for osteoclasts, osteoblasts, bone mass, and the phase portrait for scenario 5 under $\sigma =0.05$, $\sigma =0.10$, $\sigma =0.20$, and $\sigma =0.40$

Figure 10 shows the scenario 5, which exhibits one pure real negative eigenvalue and two pure imaginaries eigenvalues for $\sigma = 0.05, 0.10$. In those cases, results show a limit cycle with the pairs (osteoclast, osteoblast) for the nontrivial steady states given by (1.3171, 263.4278) and (1.5078, 301.5535) for $\sigma = 0.05, 0.10$, respectively. However, due to the original Komarova’s parameter for bone mass evolution, the bone mass is increased because of the excess of osteoblasts in both cases. For $\sigma = 0.20, 0.40$, the positive eigenvalues produce the oscillation of osteoblasts and osteoclast growing over time. Thus, it is an unstable behavior that can be seen in carcinomas (Zanoletti et al. 2015).

### Tumor with Komarova’s structure

**Fig. 11:**
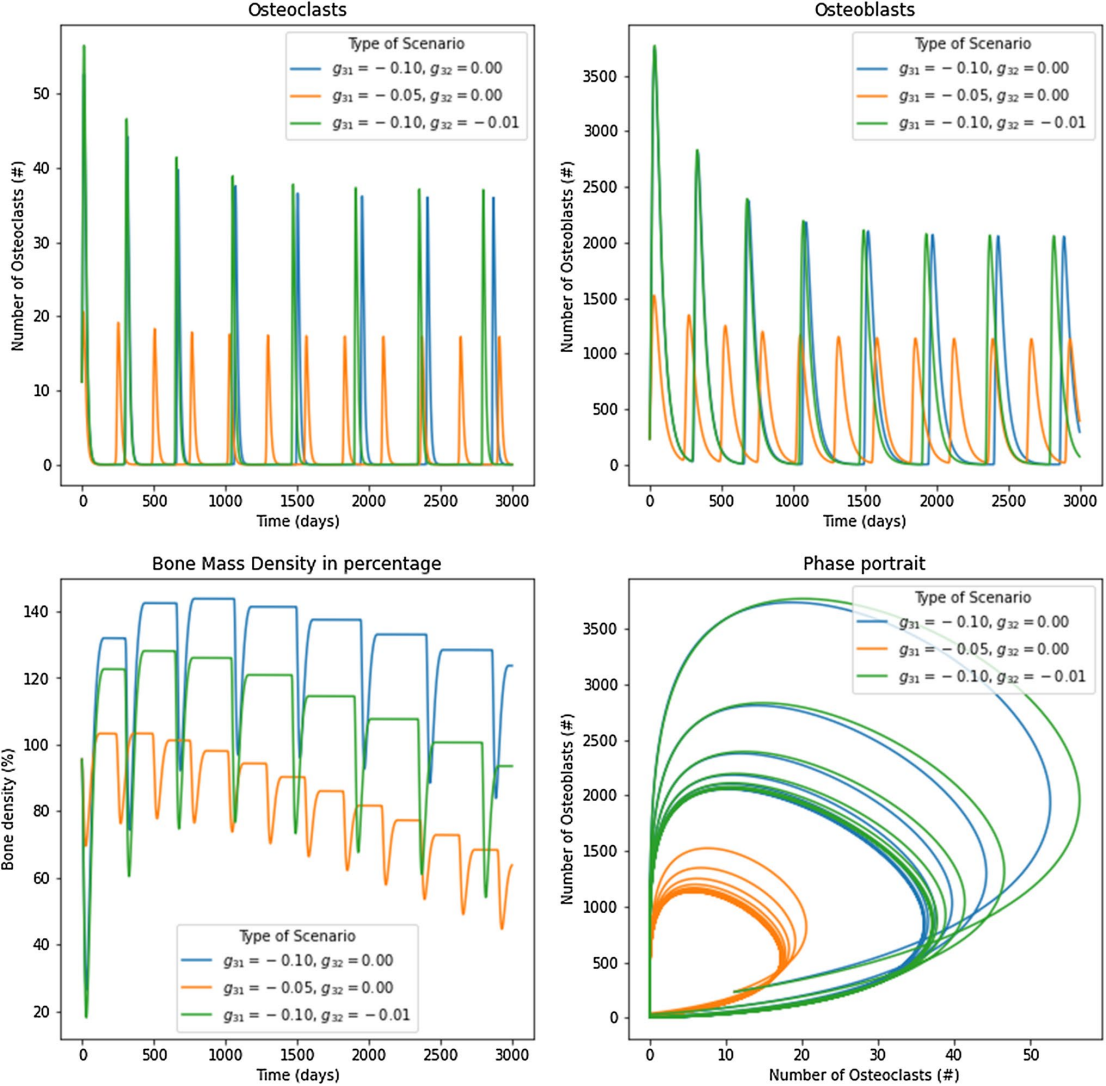
Results for osteoclasts, osteoblasts, bone mass, and the phase portrait for the second model under $\sigma =0.05$. Three scenarios were studied with different parameters following the stability analysis. In blue $g_{31}=-0.10$, $g_{32}=0.00$; in orange $g_{31}=-0.05$, $g_{32}=0.00$; and in green $g_{31}=-0.10$, $g_{32}=-0.01$

The second model presented considers the tumor cell unitary density and includes two new paracrine parameters. This parameters call $g_{31}$ and $g_{32}$ take into account how the tumor affects the evolution of osteoclasts and osteoblasts. Equations (17) show a structure similar to Komarova’s model. The solution to those equations requires that $g_{31}$ and $g_{32}$ can be picked from an infinite number of values, but using the stability analysis and selecting a few whose results are stable. In Fig. 11, results for different combinations of the paracrine factors are shown for a value of $\sigma =0.05$. For scenario with $g_{31}=-0.10$ and $g_{32}=0.00$, it is observed that the result is stable by Routh–Hurwitz conditions. It has a damped oscillation due to one pure real negative eigenvalue and two others with negative real part, with steady states given by (0.5479, 490.0362), for osteoclasts and osteoblasts, respectively. The bone mass grows in the first cycles due to the high initial value of osteoblasts, but it is reduced over time due to the decreasing of bone cells. It can be compared to the previous models, where there was a reduction in the bone remodeling activity producing a decrease in bone mass. It also shows the versatility of the first model, which does not use additional parameters. Thus, the result is similar to the structure that exhibits the myeloma (Pour Hájek et al. 2011). An infinite number of combinations can be obtained using Table 3. By using $g_{31}=-0.05$ and $g_{32}=0.00$, we observed damped oscillations with a steady state given by (0.7967, 336.9763), where an increase in osteoclasts, diminution of osteoblasts, and a high frequency in the bone remodeling cycles is observed, compared to the previous example. Finally, using parameters $g_{31}=-0.10$ and $g_{32}=-0.01$, it is possible to determine the way in which $g_{32}$ has an effect that tends to decrease the number of osteoblasts (Fig. 11).

### Tumor affecting the Komarova’s structure through the paracrine and autocrine parameters

**Fig. 12:**
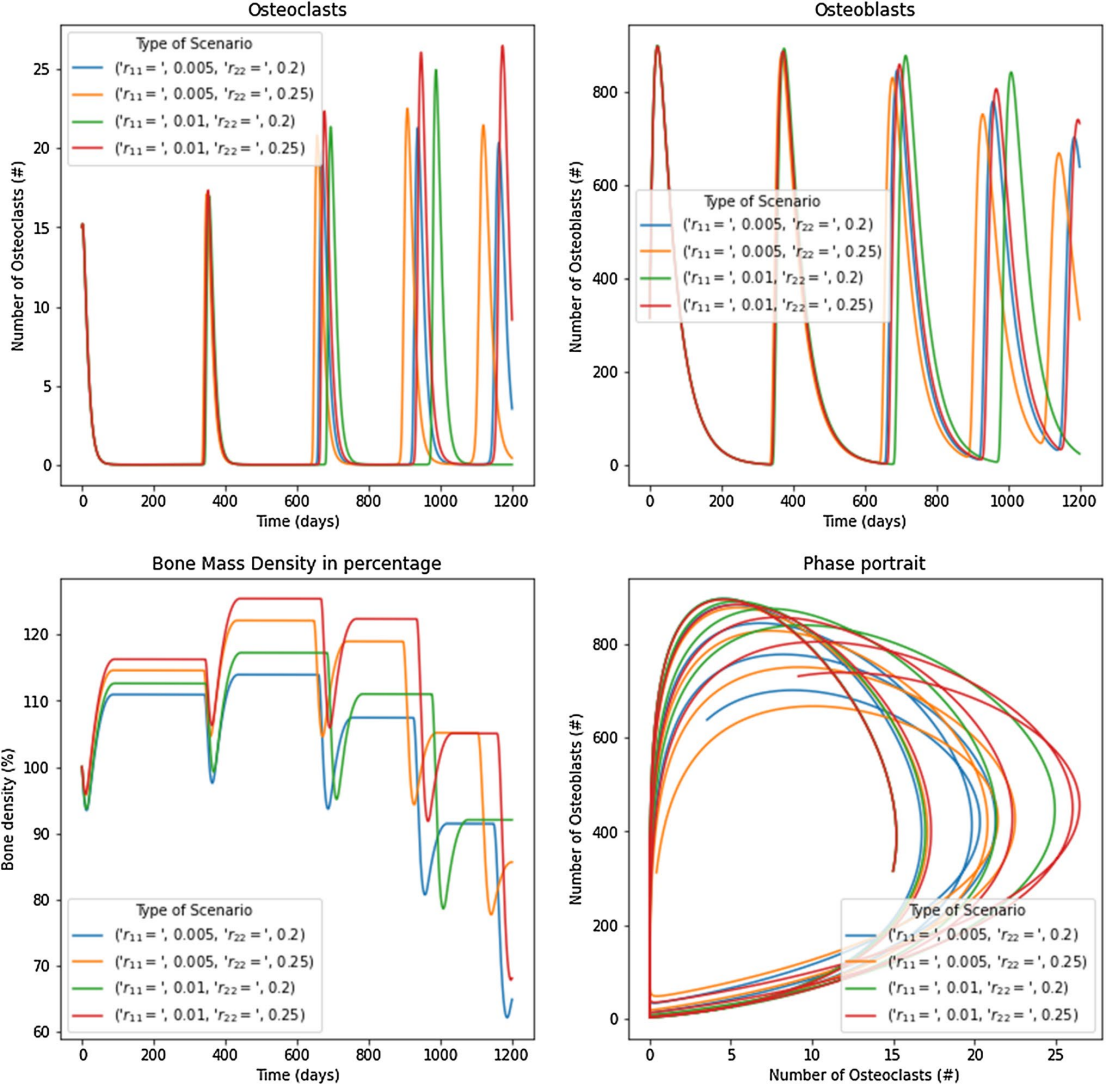
Results for osteoclasts, osteoblasts, bone mass, and the phase portrait for the second model under $\sigma =0.05$. The figure shows all the parameters used in the simulation

Ayati et al. (2010) proposed to include new parameters affecting the paracrine and autocrine values. That model was created thinking about the presence of myeloma in bone. The new model consider the fact of $r_{ij}$ as modifiers of $g_{ij}$ in function of the tumor cell density, i.e., $\widetilde{g_{ij}}=\widetilde{g_{ij}}(g_{ij},r_{ij},D)$. In the example presented in this paper, a combination of $r_{ij}$ as shown in Fig. 12 was used. The term $r_{ij}D/ \sigma$ was added to elevate or reduce the paracrine and autocrine factors in the function of tumor cells. Results showed damped oscillations, similar to models 2 and 3. Initially, the system increases the osteoclasts and osteoblasts, but over time, it is coupled with the tumor, producing a drop in the BMU cells until it achieves a steady state. $g_{12}=0=g_{21}$ were assumed for the simulations. Then, values of $g_{11}=0.005$, $g_{22}= 0.2$ were considered to obtain the steady state $\overline{x_1} = 4.9938$ and $\overline{x_1} = 315.9015$; in the second case, $g_{11}=0.005$, $g_{22}= 0.25$ were used to obtain the steady state $\overline{x_1} = 7.8122$ and $\overline{x_1} = 343.0589$; in the third case $g_{11}=0.010$, $g_{22}= 0.20$ were implemented to obtain the steady state $\overline{x_1} = 5.1403$ and $\overline{x_1} = 323.6118$; finally, $g_{11}=0.010$, $g_{22}= 0.25$ were used to obtain the steady state $\overline{x_1} = 7.668$ and $\overline{x_1} = 353.6606$. In all cases, the bone mass decreases over time as the tumor grows. It is important to remark that stability analysis can pick infinite combinations of parameters that can mimic multiple pathological scenarios.

## Conclusion

Exploring new mathematical models for predicting and studying the evolution of tumors and cancer has been widely used (Medina (2018) Altrock et al. (2015) Botesteanu et al. (2016) Simmons et al. (2017) Kimmel (2010)). The goal has been to establish as many scenarios possible for predicting the effects of tumors on different tissues. They were conceived to study the spread, growth and localization of the different tissues. They were conceived to study the spread, growth and localization of the different tissues. This paper specifically focused on the bones, where the effect of tumors on the bone remodeling cycle has been researched. In experimental and clinical investigations, observations have shown that the number of osteoclasts can increase over time. For instance, Panaroni et al. (2017) reported the imbalance between the formation and resorption produces osteolysis, which is the pathogenesis that runs into myeloma. Thus, the proliferation of osteoclasts is accompanied by a reduction in the production of osteoblasts Yang et al. (2022) Panaroni et al. (2017). Moreover, multiple myeloma is not only characterized by high blood calcium levels and fractures, but also associated with conditions such as anemia, hypercalcemia, and renal insufficiency (Yang et al. 2022). Researchers have studied bone tissue as a tumor microenvironment (Kähkönen et al. 2021). This biological and chemical environment is home to the metastatic process. The bone imbalance classifies metastasis as the osteolytic process characterized by an imbalance producing excess resorption (Kähkönen et al. 2021) due to an increase in osteoclasts or reduction in the bone remodeling process (Coleman 2006), which is common in lung and breast cancer. A positive imbalance in favor of formation is due to osteoblastic (osteosclerotic) metastasis (Coleman 2006) Kähkönen et al. (2021). This type of metastasis is common in prostate and pathological formation of new bone. Accordingly, models described herein may mimic those possibilities vis-á-vis relationship between bone remodeling process and tumor growth. Moreover, models mentioned above may help to prove scenarios and types of tumors, propose and test treatments “in silico,” and be a framework for projecting the tumor effect on the remodeling process in time for predicting the process. Therefore, these models can propose altering the cycle by promoting differentiation, reducing the frequency of remodeling, or increasing/decreasing the bone mass deposition in a mathematical laboratory. Although the exact (and numerical) relationship between tumor and bone remodeling process is unknown, this paper proposes, analyzes and compares three approaches to bone remodeling - tumor coupling. In the first model, the factor $(1-D)$ is located as a numerator (decreasing the effect) or denominator (increasing the impact) of the proliferation (for $\alpha _i$-parameters) and removal (*β* − *j*-parameters) terms. The model can reproduce the increase in osteoclasts in scenario 3, similar to the multiple myeloma or osteolysis processes reported Kähkönen et al. (2021) Coleman (2006). By contrast, a positive imbalance in bone formation is presented in scenario 6, similar to osteosclerotic diseases. Moreover, scenarios 1, 4, 7, and 8 showed different possible disorders associated with bone remodeling processes. But overall, the remodeling process is lost due to the tumor’s presence. The second model includes the tumor cell density as a factor in the formation of osteoblasts and osteoclasts, which is influenced by paracrine factors called $g_{31}$ and $g_{32}$. This model can also reproduce the frequency formation change of the bone remodeling process due to the tumor and produce a reduction in bone formation due to the relative imbalance between osteoclasts and osteoblasts. Similarly, the third model, reported by Ayati et al. (2010), can reproduce multiple myeloma. Overall, we have described models ranging from the lowest to the greatest number of parameters used in equations. The first and second models can be used for modeling the tumor effect on the remodeling process for osteosclerotic and osteolytic processes. The third model is a good choice for modeling a complex process like multiple myeloma. In the future, the coherence and usability of these models should be studied in cancer treatments as well as in other environmental factors such as hypoxia, metabolic defects, and growth/aging processes.

A recent model has been developed by Rapisarda et al. (2019), where a coupling for the mechanical and biological modeling of bone is presented in a way that the stimulus for this dynamic system is included, with a porosity factor and a differentiating form of osteoblasts and osteocytes (Corte et al. 2020). Some suggestions for future works are the improvement in the signaling and stimulus for the bone remodeling process, which supports the objective of this paper. Some other recent works include a not so well understood dynamics of the bone remodeling under metabolic diseases with today’s treatments. One of these is the one done by Kameo et al. (2020), where an in silico model was developed to understand how a perturbation on a specific molecule produce an effect on the bone metabolic dynamics over time, which provides a powerful tool for predicting certain drug effects on the metabolic bone dynamics for an specific disease. Oumghar et al. (2020) provide a technical review of these sort of models, aiming to provide a timely and critical analysis of the models developed for the bone remodeling process, focusing in models that address bone diseases. Suggestions from these authors is to develop new models for 1D or 2D geometries, to represent the BMU evolution of damaged bone.

Despite the essential insights that this research can give to the academic community dedicated to the cancer research, we should mention some limitations that have to be considered in future works. First of all, the model herein consigned is based on the Komarova’s one (Komarova et al. 2003) which is an abstraction of the reality and the parameter referenced therein are obtained from Stability analysis. Those parameter respond to and fit the number of cells involved into the basic multicellular unit, i.e., initial number of osteoblasts and osteoclasts, the bone oscillation, and the period of time for mass replacement, Komarova et al. could formulate a mathematical model and the parameters could represent the experimental observation. Employing a similar approach, the modification of the Komarova’s seminal model was made using parameters that come from the stability analysis. Thus, future works must contrast the mathematical model (and the parameters) and the experimental evidence. It is important to remark that the exact relationship between the tumor growth and bone remodeling process is unknown. Hence, different metabolic processes and other mechanical conditions can modify the BMU, making the result of these models diverge from the actual remodeling process behavior. In future works, the model should be extended to include mechanical environment and the drugs that can be used for improving the bone mass or reduce their quality. This would require extending the model to a continuum one where this dynamic process would resolve around the BMU. Each of the modifications should be compared with actual tumor diseases and other techniques as statistics and machine learning could help us for identifying the model variations and its parameters. Another limitation lies in the variations. In this paper we proposed three plausible possibilities from a biological point of view, as previously described. However, all the potential verification to the model or, even to change the Komarova’s model as a paradigm in the BMU representation, must be verified. In future works, we will use the models herein proposed to represent in the continuum (2D and 3D) the effect of the mechanical loads into the BMU and their relationship with the bone mass quality. The challenge will be to find a way to include stress and strains in Komarova’s model, and how Komarova’s model can modify the macrostructure of the bone, i.e., we will propose a multiscale model for representing tumor growth in bone.

**Fig. 13:**
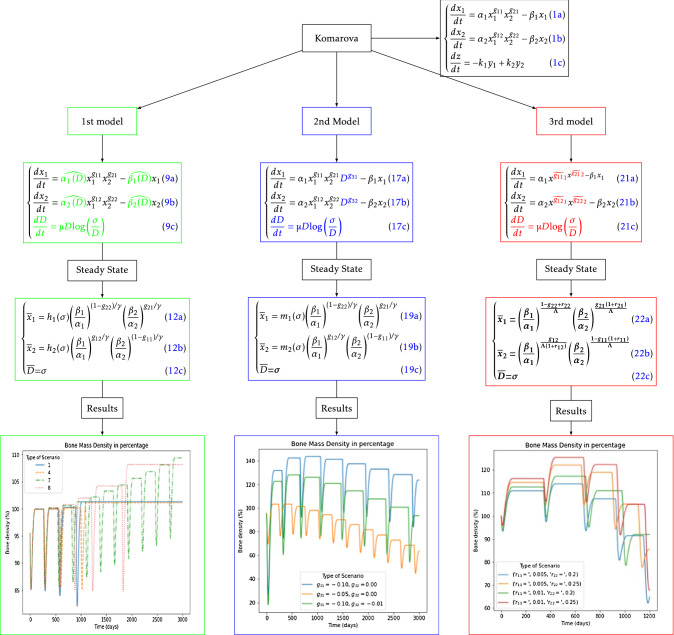
Diagram for a quick comparison between the models and the results obtained

Finally, Fig. 13 shows the entire process with details. At the top of the diagram the Komarova’s base model is shown with its equations. From this model, a first alternative for coupling the tumor with a “damage” parameter is shown where variations of the base model are colored with green for the second differences with Komarova’s base model are shown in blue and in the third one with red. In the following part, the final equations from stability analysis are shown and then the results obtained from said equations, in which the temporal behavior is shown for the bone mass density in several scenarios. This is a schematic representation of the entire process with the main steps and results obtained.

## Data Availability

The data obtained on the solution of these models can be accessed by sending an email to the corresponding author.
